# Transcription coactivator Cited1 acts as an inducer of trophoblast-like state from mouse embryonic stem cells through the activation of BMP signaling

**DOI:** 10.1038/s41419-018-0991-1

**Published:** 2018-09-11

**Authors:** Yanli Xu, Xinlong Luo, Zhuoqing Fang, Xiaofeng Zheng, Yanwu Zeng, Chaonan Zhu, Junjie Gu, Fan Tang, Yanqin Hu, Guang Hu, Ying Jin, Hui Li

**Affiliations:** 1 Basic Clinical Research Center, Renji Hospital, Department of Histology, Genetics and Developmental Biology, Shanghai Key Laboratory of Reproductive Medicine, Shanghai JiaoTong University School of Medicine, 225 South Chongqing Road, 200025 Shanghai, China; 2 CAS Key Laboratory of Tissue Microenvironment and Tumor, Shanghai Institute of Nutrition and Health, CAS Center for Excellence in Molecular Cell Science, Shanghai Institutes for Biological Sciences, University of Chinese Academy of Sciences, Chinese Academy of Sciences, 320 Yueyang Road, 200032 Shanghai, China; 3 Epigenetics and Stem Cell Biology Laboratory, National Institute of Environmental Health Sciences, Research Triangle Park, NC, 27709 USA; 4 Present Address: KU Leuven Department of Development and Regeneration, Stem Cell Institute Leuven, Herestraat 49, 3000 Leuven, Belgium

## Abstract

Trophoblast lineages, precursors of the placenta, are essential for post-implantation embryo survival. However, the regulatory network of trophoblast development remains incompletely understood. Here, we report that Cited1, a transcription coactivator, is a robust inducer for trophoblast-like state from mouse embryonic stem cells (ESCs). Depletion of *Cited1* in ESCs compromises the trophoblast lineage specification induced by BMP signaling. In contrast, overexpression of *Cited1* in ESCs induces a trophoblast-like state with elevated expression of trophoblast marker genes in vitro and generation of trophoblastic tumors in vivo. Furthermore, global transcriptome profile analysis indicates that ectopic *Cited1* activates a trophoblast-like transcriptional program in ESCs. Mechanistically, Cited1 interacts with Bmpr2 and Smad4 to activate the Cited1–Bmpr2–Smad1/5/8 axis in the cytoplasm and Cited1–Smad4–p300 complexes in the nucleus, respectively. Collectively, our results show that Cited1 plays an important role in regulating trophoblast lineage specification through activating the BMP signaling pathway.

## Introduction

The specification of extraembryonic trophectoderm (TE) and inner cell mass (ICM) at E3.5 is the first cell fate decision of mammalian development^1,2^. TE cells give rise to trophoblast lineages, thereafter mediating implantation and generating the functional placenta^3^. Given the indispensable role of the trophoblast for embryo development, a great deal of effort has been made to unravel the regulatory networks of trophoblast development.

Embryonic stem cells (ESCs) and trophoblast stem cells (TSCs), which are derivatives of ICM and TE respectively, retain the capacity to self-renew indefinitely and model their counterparts in vivo functionally^4–6^. ESCs are generally considered to have a weak ability to generate trophoblast lineages spontaneously due to their ICM origin^7^. Nonetheless, it was found that mouse ESCs can become trophoblast-like cells by forced expression of key trophoblast-associated factors such as *Cdx2*^8,9^, *Gata3*^10^, *Arid3a*^11,12^, or *Brog5*^13^. Moreover, depletion of *Oct4*, an ESC core transcription factor^14^, leads to the commitment of ESCs into trophoblast-like cells^15,16^. The similar phenotype was observed in *Tet1*-depleted ESCs^17^. In addition, signaling pathways contribute to the ESC fate determination. BMP4 signaling was reported to enable ESCs to become trophoblast-like cells robustly^18–29^. BMP and Activin/Nodal/TGF-β signaling pathways are two branches of the TGF-β superfamily, which use different sets of receptors and Smad transducers (Smad1/5/8 for BMP signaling and Smad2/3 for TGF-β signaling). These two branches often antagonize each other due to the competition for the common Smad4^30^. BMP signaling also plays crucial roles for trophoblast and placenta development^31^. However, the intracellular factors that can regulate its activity, thereby controlling trophoblast differentiation, remain to be discovered.

Analysis of published transcriptional datasets led us to identify Cited1 as a robust inducer of trophoblast-like state from mouse ESCs. Cited1 (Cbp/p300 Interacting Transactivator with Glu/Asp Rich Carboxy-Terminal Domain 1), formerly known as Msg1^32^, was reported to interact with Smad4^33,34^, estrogen receptor alpha and beta^35^, and Cbp/p300^35^. In addition, Cited1 could positively regulate TGF-β and BMP signaling through its association with the Smad/p300/Cbp-mediated transcriptional complex in melanoma and metanephros cells^34,36^. During development, Cited1 plays a role in cell growth, pigmentation of melanocytes, early nephronic patterning and tumorigenesis^32,34,37–39^. It also contributes to the terminal maturation of trophoblast subtypes and the organization of placenta structure in the mouse^40^. Nonetheless, the precise role of Cited1 in the early stage of trophoblast development remains elusive.

Here, we investigate the role of Cited1 in ESC fate determination. Depletion of *Cited1* dramatically compromises the capacity of ESCs to become trophoblast-like cells induced by BMP4. In contrast, ectopic *Cited1* expression induces ESC trans-differentiation into trophoblast-like cells under the self-renewal culture condition and trophoblastic tumors with internal hemorrhage in vivo. Global transcriptional analysis shows that ectopic *Cited1* expression initiates a trophoblast-like transcriptional program in ESCs. Mechanistically, Cited1 can associate with Bmpr2 in the cytoplasm to enhance the phosphorylation of Smad1/5/8 and with Smad4 in the nucleus to enhance its transcriptional activity, respectively. Therefore, Cited1 could trigger a transition of ESCs from a self-renewal state to a trophoblast-like fate through activating the BMP signaling pathway.

## Results

### Cited1 is highly expressed in trophoblast lineages in vitro and in the trophectoderm of early mouse embryos

**Fig. 1:**
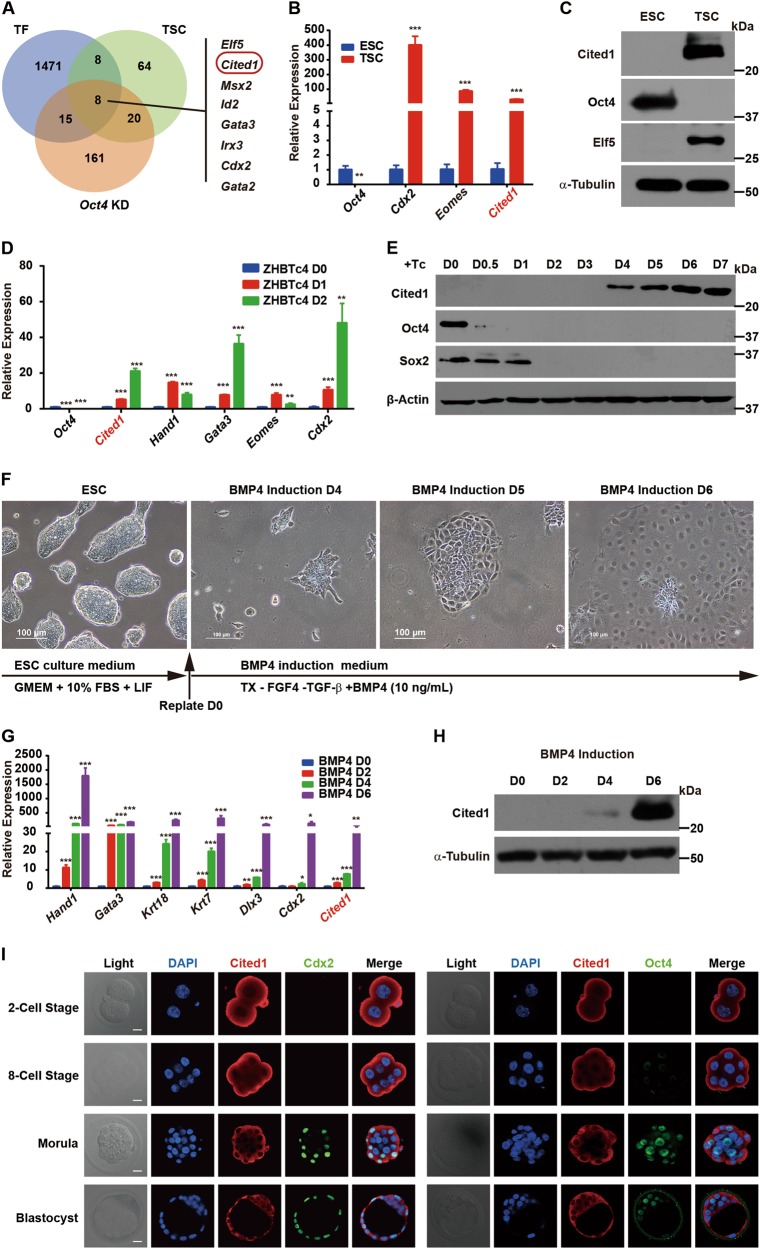
*Cited1* is highly expressed in cultured trophoblast lineages and in the trophectoderm of early mouse embryos **a** A venn diagram showing the intersections of 3 gene sets: highly differentially expressed genes (DEGs) in TSCs versus ESCs (TSC, green), DEGs upon *Oct4* knockdown (*Oct4* KD, pink) and transcription factors (TF, blue). The number of genes is indicated. **b** Expression patterns of *Cited1* and marker genes related to pluripotency and trophoblast lineage in E14T ESCs and TSCs, examined by qRT-PCR analysis. The average mRNA level in ESCs was set at 1.0. Data are shown as mean ± SD (*n* = 3). **p < 0.01, ***p < 0.001. **c** Representative western blot result of Cited1, Oct4 and Elf5 in ESCs and TSCs. α-Tubulin was used as a loading control. **d** Expression levels of *Cited1* and trophoblast markers during differentiation of the ZHBTc4 ESCs were determined by qRT-PCR analysis. The average mRNA level in ZHBTc4 cells cultured without Tc was set at 1.0. Data are shown as mean ± SD (*n* = 3). **p < 0.01, ***p < 0.001. **e** Representative western blot result of Cited1, Oct4, and Sox2 during differentiation of ZHBTc4 ESC cells, indicated as days after addition of tetracycline (Tc). β-Actin was used as a loading control. **f** The morphological changes of ESCs in response to BMP4 and schematic representation of the strategy to induce trophoblast trans-differentiation from ESCs by BMP4 treatment. Scale bar: 100 μm. **g** Expression levels of *Cited1* and trophoblast markers during ESC trans-differentiation towards trophoblast lineages upon BMP4 induction were determined by qRT-PCR analysis. The average mRNA level in E14T ESCs without BMP4 treatment was set at 1.0. Data are shown as mean ± SD (*n* = 3). *p < 0.05, **p < 0.01, ***p < 0.001. **h** A representative western blot result of Cited1 protein levels during BMP4-induced ESC trans-differentiation to trophoblast lineages. α-Tubulin was used as a loading control. **i** Expression patterns of Cited1 in early embryos examined by immunofluorescence staining. Scale bar: 20 μm

To identify transcription-related factors involved in the early TE formation during mouse embryonic development, we analyzed published microarray data of ESCs, TSCs, and TSC-like cells derived by *Oct4* knockdown (KD) in ESCs^10,12^. We compared 3 sets of genes, including top 100 genes highly expressed in TSCs versus ESCs, top 1% of upregulated genes upon *Oct4* KD in ESCs and 1502 transcription-associated factors from a commercial library (Table S1) and found that 8 genes were shared by all 3 gene sets. They were *Cited1*, *Irx3*, *Msx2* and known TE lineage markers *Elf5*, *Cdx2*, *Gata3*, *Gata2*, and *Id2* (Fig. 1a). *Cited1* was chosen for further investigation, since its knockout (KO) mice showed placenta defects^40^ and its function in ESC fate determination remained unclear.

We began with examining the expression pattern of Cited1 and found that its transcript and protein levels were significantly higher in TSCs than in ESCs, having a similar pattern to known trophoblast markers (*Cdx2*, *Eomes*, or Elf5) (Fig. 1b, c). In contrast, Oct4 was only found in ESCs. We further determined Cited1 expression profile in two trophoblast induction models. First, *Oct4* was depleted in the ZHBTc4 mouse ESC line, in which the endogenous *Oct4* loci were replaced with tetracycline-regulated *Oct4* transgene^15^. Cited1 expression was induced by *Oct4* KD, together with upregulation of known trophoblast markers, such as *Cdx2*, *Gata3*, and *Hand1* (Fig. 1d, e). Second, Cited1 expression was examined during BMP4-induced trophoblast differentiation from ESCs. With the addition of BMP4, ESC colonies were gradually converted to flattened epithelia with a cobblestone appearance at day 6 (Fig. 1f). The effective trophoblast induction was further indicated by increased expression of trophoblast markers (Fig. 1g). Krt7, a pan- trophoblast marker, was expressed in the majority of BMP4-induced cells (Figures S1A-S1C). Moreover, teratomas generated from BMP4-treated cells contained typical structures of trophoblastic hemorrhagic lesions with large amounts of trophoblastic giant cells surrounding blood-filled lacunas (Figure S1D). Importantly, Cited1 was also significantly upregulated by BMP4 treatment (Fig. 1g, h). Therefore, results from both models suggest the close association of Cited1 with trophoblast lineage induction.

Additionally, we found that the expression level of *Cited1* increased gradually during TSC differentiation induced by FGF4 withdrawal^6^, in a manner similar to pan-trophoblast markers (*Dlx3*, *Krt7*, and *Psx1*) but different from TSC markers (*Elf5*, *Cdx2* and *Eomes*) (Figure S2A). The finding indicates that Cited1 might also function in differentiated trophoblast cells.

At last, we determined the expression of Cited1 in early mouse embryos from the 2-cell to blastocyst stage by immunofluorescence staining (Fig. 1i). Cited1 was detectable in all these stages and primarily localized in the cytoplasm. Interestingly, Cited1 was mostly found in the outer layer cells, if any in the inner cells, of embryos at 8-cell and morula stages. At the blastocyst stage, Cited1 was expressed with a higher level in TE cells than in ICM cells. As controls, Cdx2 was found in the nucleus of TE cells in blastocysts, while Oct4 and Cnot3 (a known cytoplasmic protein)^41^ were mainly detected in the nucleus and cytoplasm of ICM cells, respectively (Fig. 1i and S2B). Taken together, Cited1 is highly expressed in the cells of trophoblast lineages both in vitro and in vivo, suggesting its potential role for trophoblast development.

### *Cited1* depletion compromises BMP4-induced trophoblast differentiation

**Fig. 2:**
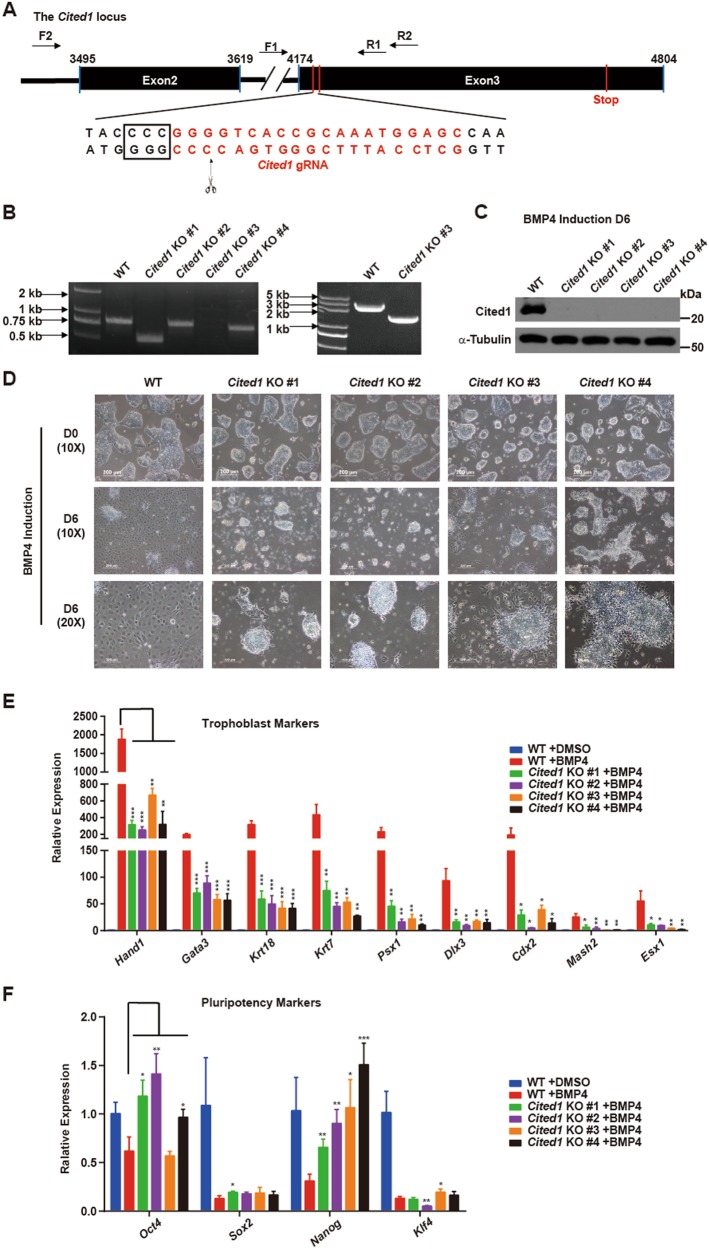
*Cited1* depletion compromises BMP4-induced trophoblast conversion from ESCs. **a** Schematic representation of the strategy for *Cited1* knockout (KO) by the CRISPR/Cas9 approach. The PAM sequences are in the rectangle. The cleavage site is pointed out by the scissor and arrow. Positions of the designed primers for genomic PCR are shown as arrows. The sequence of gRNA is shown in red color. **b** Four *Cited1* KO E14T ESC cell lines with frame-shifted- or large fragment deleted-*Cited1* were identified. This panel shows the genomic DNA PCR results. **c** Western blot analysis of protein levels of Cited1 in the parental wild-type (WT) ESCs and four *Cited1* KO ESC lines after treatment with BMP4 for 6 days. **d** Morphology changes of parental wild-type (WT) ESCs and four *Cited1* KO ESC lines before (top panel) and after (middle and bottom panels) treatment with BMP4 for 6 days. Scale bar: 200 μm (top and middle panels); 100 μm (bottom panel). **e**, **f** qRT-PCR analysis for expression levels of trophoblast (**e**) and pluripotency (**f**) markers in the four *Cited1* KO ESC lines and their parental WT counterparts after treatment with BMP4 for 6 days. The comparison was made between *Cited1* KO and WT ESCs at day 6 upon BMP4 treatment. The average mRNA level in untreated WT ESCs was set at 1.0. Data are shown as mean ± SD (*n* = 3). *p < 0.05, **p < 0.01, ***p *<* 0.001

To test the function of Cited1 in trophoblast induction from ESCs, we generated *Cited1* KO ESCs using the CRISPR/Cas9 system^42^. A construct containing sequences of Cas9, a puromycin-resistant cassette and a gRNA targeting the third exon of *Cited1* was transfected into ESCs (Fig. 2a). Six resistant colonies were picked and validated by genomic DNA PCR and Sanger sequencing. Biallelic deletion of *Cited1* was verified in all six clones, four of which contained frame-shift mutations (KO #1, KO #2, KO #4) or large fragment deletion (KO #3) (Fig. 2b and S3). Western blotting analysis further validated deletion of *Cited1* in these clones (Fig. 2c), which were then utilized to determine whether Cited1 would play a role in BMP4-induced trophoblast differentiation.

Wild-type (WT) ESCs displayed typical trophoblast-like morphology at day 6 of BMP4 treatment, with significant upregulation of trophoblast genes (*Hand1*, *Gata3*, *Cdx2*, *Krt7*, *Krt18*, *Psx1*, *Dlx3*, *Esx1*, and *Mash2*) and downregulation of pluripotency genes (*Oct4*, *Sox2*, *Nanog*, and *Klf4*). However, *Cited1* KO compromised the trophoblast conversion. BMP4-induced cobblestone-like morphology in WT ESCs was not observed in *Cited1* KO cells (Fig. 2d). At molecular levels, dramatically elevated expression of trophoblast marker genes in BMP-treated cells was significantly reduced by *Cited1* deletion (Fig. 2e). Of note, *Cited1* depletion attenuated the BMP4-induced downregulation of *Oct4* and *Nanog*, whereas *Sox2* and *Klf4* remained down (Fig. 2f). The phenomenon suggests that Cited1 could be only involved in the regulation of a subset of pluripotency markers during this differentiation process. These results indicate an indispensable role of Cited1 in trophoblast differentiation.

### Forced expression of *Cited1* activates trophoblast gene expression

**Fig. 3:**
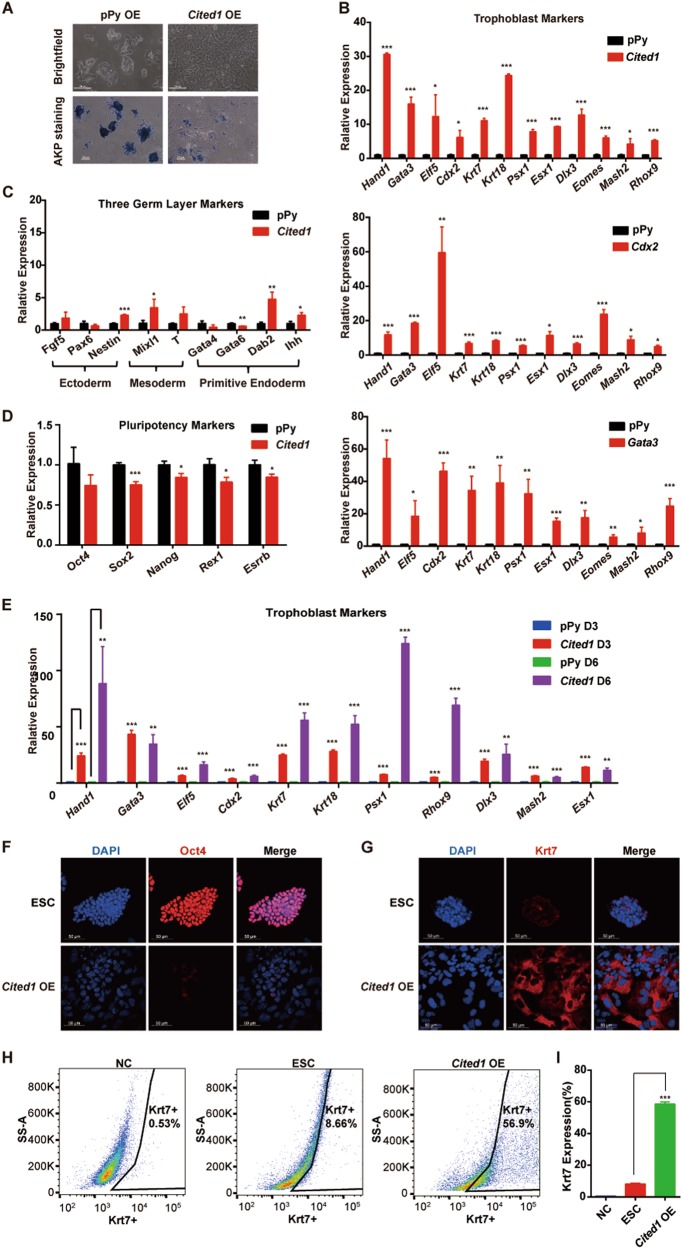
Ectopic *Cited1* activates trophoblast lineage genes. **a** Typical morphological changes and AKP staining in E14T ESCs after overexpressing *Cited1* for 2 days. Scale bar: 100 μm. **b** Results of qRT-PCR analysis of expression levels of trophoblast markers in ESCs overexpressing *Cited1*, *Cdx2*, or *Gata3* for 3 days. The average mRNA level in cells transfected with the control vector pPy was set at 1.0. Data are shown as mean ± SD (*n* = 3). *p *<* 0.05, **p < 0.01, ***p < 0.001. **c**, **d** Results of qRT-PCR analysis of expression levels of three germ layer markers (**c**) and pluripotency-associated markers (**d**) in ESCs overexpressing *Cited1* for 3 days. The average mRNA level in cells transfected with the control vector pPy was set at 1.0. Data are shown as mean ± SD (*n* = 3). *p *<* 0.05, **p < 0.01, ***p < 0.001. **e** qRT-PCR analysis of expression levels of trophoblast markers after transfection of plasmids as indicated in ESCs over a time course. The average mRNA level in cells transfected with the control vector pPy was set at 1.0. Data are shown as mean ± SD (*n* = 3). **p < 0.01, ***p < 0.001. **f**, **g** Immunofluorescence staining of ESCs after transfection of *Cited1* for 6 days. Samples were stained with anti-Oct4 antibody (red) (**e**) and anti-Krt7 antibody (red) (**f**), respectively. DAPI staining highlights the nuclei (blue). Scale bar: 50 μm. **h** Flow cytometry density plots for Krt7 expression in ESCs after transfection of *Cited1* for 6 days. Cells were fully dispersed, fixed, and immunostained for Krt7. For the negative control (NC), cells were exposed only to secondary antibody without prior exposure to the primary Krt7 antibody. **i** The statistical analysis of flow cytometry data for percentages of cells positive for Krt7 expression in ESCs overexpressing *Cited1* or an empty vector for 6 days. Data are shown as mean ± SD (*n* = 3). ***p < 0.001

We then asked whether forced expression of *Cited1* would be able to induce ESCs to trans-differentiate into a trophoblast state. To test the possibility, *Cited1* was overexpressed in ESCs under a self-renewal condition with serum and leukemia inhibitory factor (LIF) (Figure S4A). Two days after transfection, the majority of *Cited1*-transfected cells lost the doomed morphology with negative or significantly reduced intensities of alkaline phosphatase (AKP) staining, an indicator of the undifferentiated state (Fig. 3a). These cells had significantly higher levels of trophoblast genes such as *Gata3*, *Hand1*, *Elf5*, *Cdx2*, *Krt7*, *Krt18*, *Psx1*, *Esx1*, *Dlx3*, *Eomes*, *Mash2*, and *Rhox9* (Fig. 3b, upper panel). As positive controls, *Cdx2* and *Gata3*, which are known to efficiently induce trophoblast differentiation^9,10^, were also overexpressed (Fig. 3b, middle and bottom panels). The expression of trophoblast markers was comparable between *Cited1*-overexpressing cells and positive control cells. In contrast, the expression of three germ layer (ectoderm, mesoderm and endoderm)-associated genes was only slightly or not altered (Fig. 3c), suggesting that Cited1 specifically activated the trophoblast lineage markers. Moreover, pluripotency-associated markers decreased mildly in *Cited1*-transfected cells (Fig. 3d), implicating that Cited1 might disrupt ESC self-renewal through the direct activation of trophoblast genes rather than repression of pluripotency genes. Additionally, *Cited1*-transfected cells had significantly enhanced cell growth rates (Figure S4B), in line with previous reports in Wilms’ tumor and intestinal tumor cells^38,39^. To exclude the possibility that the phenotype caused by *Cited1* overexpression was ESC line dependent, same experiments were performed in another mouse ESC line (CGR8 ESC line) and consistent results were obtained (Figures S4C-S4E). Furthermore, we characterized the *Cited1-*overexpressing cells with a prolonged culture up to day 6 and determined the expression of pluripotency and lineage markers. Among the trophoblast markers tested, the majority solely mark trophoblast lineages, including *Elf5*, *Psx1*, *Esx1*, *Dlx3*, *Mash2*, and *Rhox9*, while some markers are known to also express in mesendodermal cells, such as *Gata3*, *Hand1*, and *Cdx2*. To test the possibility of mesendoderm induction by *Cited1* overexpression, we examined the expression of additional mesendoderm markers (*Lhx1, Cxcr4, Nodal, Runx2, Gsc, Pitx2, Fgf8*, and Wnt3) besides *T* and *Mixl1*. Our result revealed that *Cited1* overexpression gradually and robustly activated trophoblast marker expression with only mild or moderate alterations in three germ layer- associated genes (Figures S4F and Fig. 3e). Immunofluorescence staining results also indicated decreased and enhanced levels of Oct4 and pan-trophoblast marker Krt7, respectively, at day 6 after *Cited1* transfection (Fig. 3f, g), while the key mesodermal marker (T) was not induced at all (Figure S4G). In particular, FACS analysis revealed high percentages of Krt7^+^ cells (about 56%) in *Cited1-*overexpressing cells (Fig. 3h, i). Together, forced *Cited1* expression activated trophoblast genes and induced ESCs to exit from a self-renewal state. We next tested whether *Cited1* overexpression could cause similar phenotypes in the absence of LIF, a differentiation condition. Similar results were obtained (Figures S5A-S5E). Thus, Cited1 could activate trophoblast gene expression under both self-renewal and differentiation culture conditions.

Finally, we determined which region (s) of Cited1 conferred the ability to induce the trophoblast genes in ESCs. Cited1 protein contains the SID (Smad Interaction Domain) domain responsible for its interaction with Smad4 in the N-terminus, the conserved CR1 region in the middle, and the conserved CR2 region serving as the transactivation domain for its interaction with other factors in the C-terminus^43^. We constructed *Cited1* truncation mutants (Figure S5F) and transfected them into ESCs. Cell morphology and marker gene expression revealed that deletion of the CR2 region from the full length or presence of CR2 region alone abolished the capacity of Cited1 to activate trophoblast genes (Figures S5G and S5H), suggesting that the CR2 region was indispensable for Cited1 to execute its function in ESCs, although the region alone was not sufficient to be functional.

### Teratomas derived from *Cited1*-overexpressing ESCs exhibit trophoblastic features

**Fig. 4:**
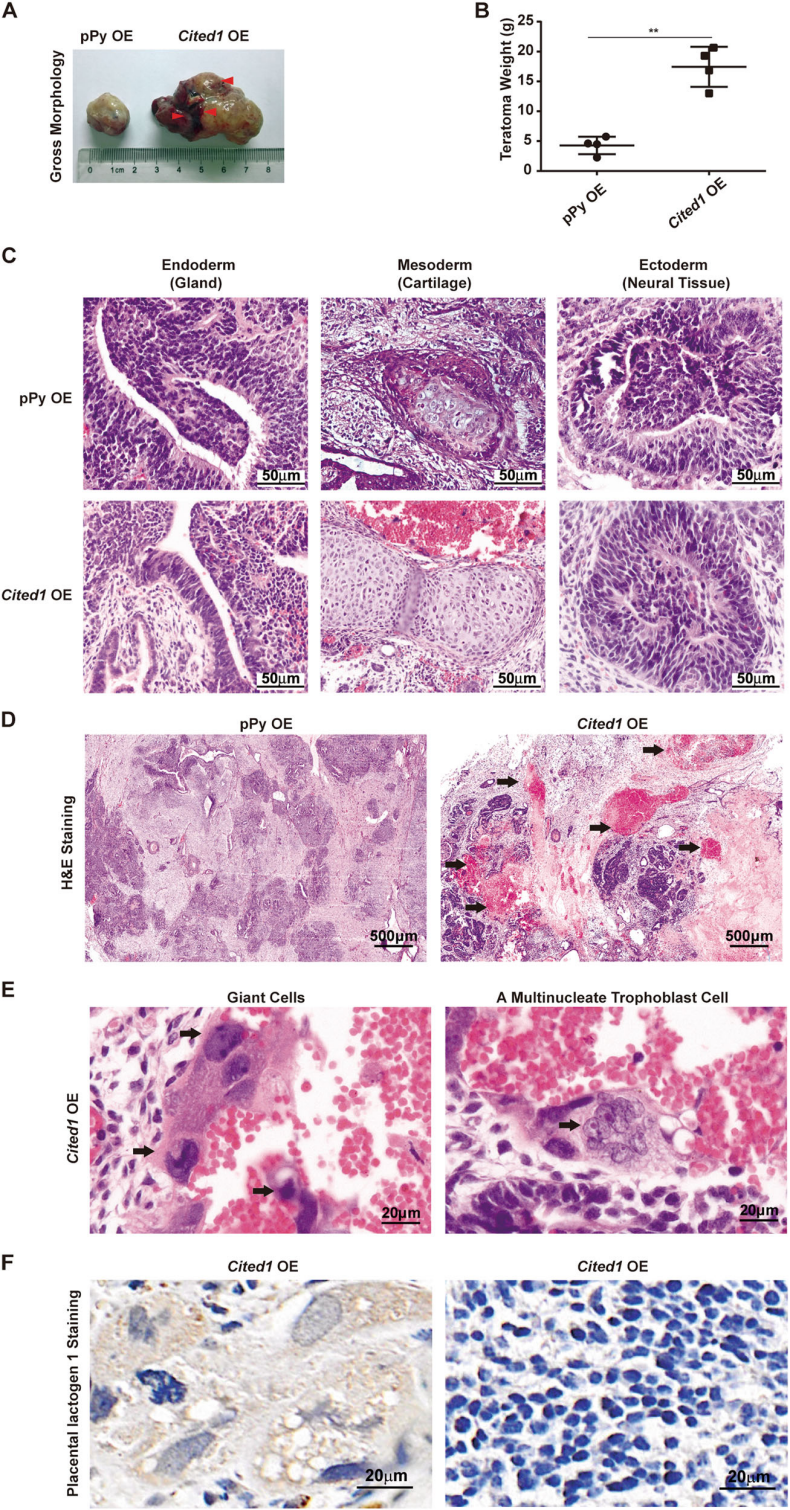
Teratomas derived from *Cited1*-overexpressing ESCs contain trophoblastic hemorrhages. **a** The gross appearance of teratomas derived from control cells or *Cited1*-overexpressing (OE) ESCs. **b** The net weight of teratomas derived from control cells and *Cited1*-overexpressing ESCs. Data are shown as mean ± SD (*n* = 4). **p *<* 0.01. **c** Hematoxylin and eosin staining (H&E) for histological sections of teratomas derived from control cells (upper panel) or *Cited1*-overexpressing ESCs (lower panel), indicating the presence of tissues and cell types from three germ layers. Scale bar: 50 μm. **d** Cross-section of H&E staining for teratomas derived from control cells or *Cited1*-overexpressing ESCs. Teratomas from *Cited1*-overexpressing ESCs contained numerous hemorrhagic loci, which are indicated by black arrows. Scale bar: 500 μm. **e** H&E staining images for sections of a teratoma derived from *Cited1*-overexpressing ESCs. The trophoblast giant cells with the enlarged nuclei (arrows) and a multinucleate trophoblast cell (with an arrow) are indicated. Scale bar: 20 μm. **f** Anti-Placental lactogen 1 immunohistochemistry staining images for sections of a teratoma derived from *Cited1*-overexpressing cells. Scale bar: 20 μm

To test the differentiation potential of *Cited1*-transfected cells in vivo, we injected the cells subcutaneously into NOD/SCID mice for teratoma formation. Cells transfected with an empty vector were used as a control. Teratomas generated from *Cited1*-overexpressing cells (referred as to *Cited1* OE teratomas) were aggressive, being about 4-fold bigger and heavier than those from control ESCs (Fig. 4a, b). Histologic analysis of teratoma sections showed that cell types from all three germ layers could be identified in both groups of teratomas (Fig. 4c). However, only *Cited1* OE teratomas contained evident and numerous internal hemorrhage foci (Fig. 4a, d), with 92 out of 100 randomly observed sections containing such blooding regions. Moreover, large numbers of clustered or scattered giant cells with a characteristic of the large cytoplasm and nuclei could be easily observed in these regions (Fig. 4e, left image). Multinucleated trophoblast cells could also be found (Fig. 4e, right image). In addition, immunohistochemical analysis with antibodies against Placental lactogen 1, a specific marker of giant cells, further validated the presence of giant cells (Fig. 4f, left image). Therefore, *Cited1*-transfected ESCs were able to generate trophoblastic teratomas in vivo.

### Overexpression of *Cited1* induces a trophoblast transcriptional program

**Fig. 5:**
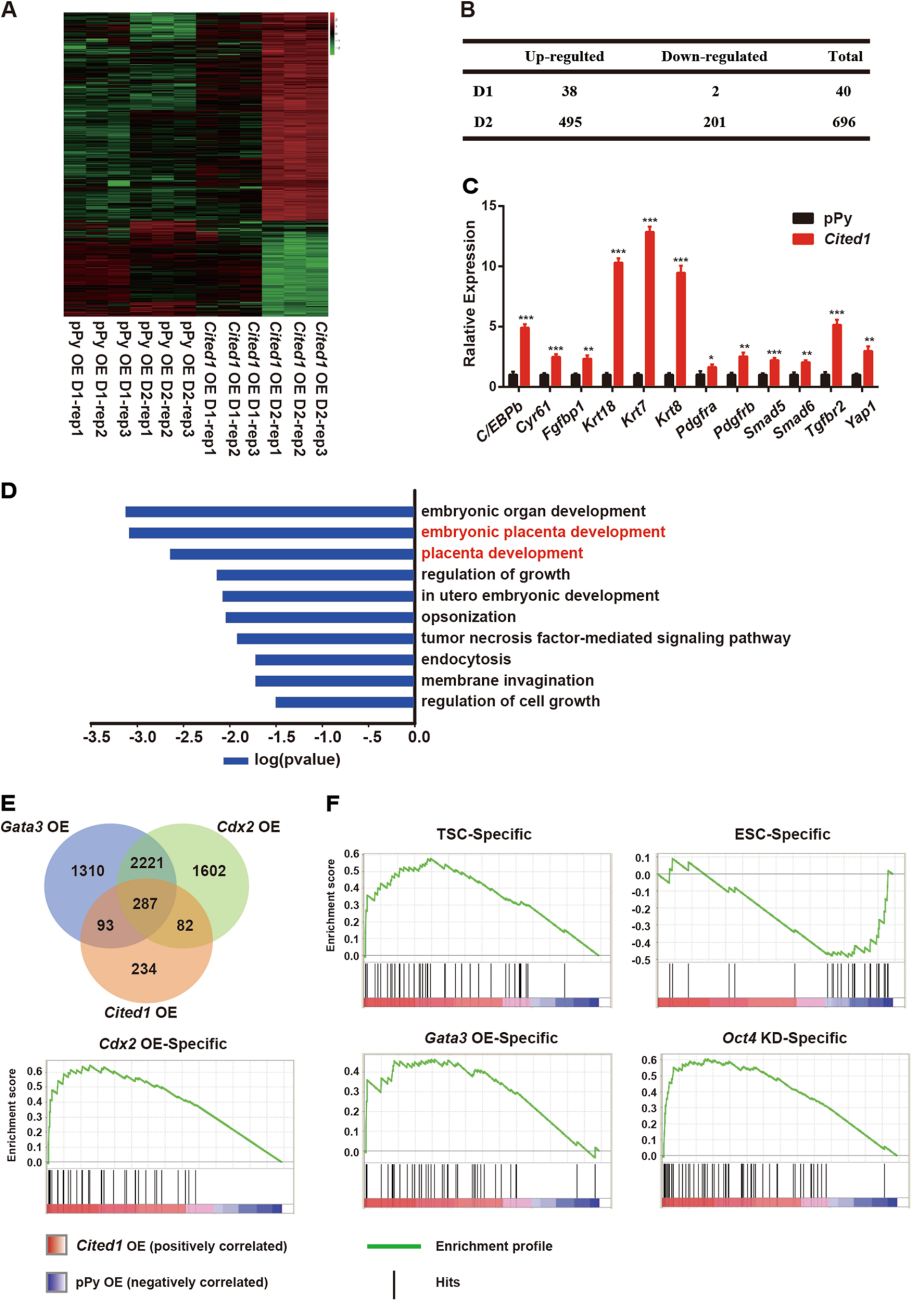
*Cited1* overexpression initiates a global trophoblast transcriptional program. **a** A heatmap of differentially expressed genes (DEGs) induced by *Cited1* overexpression in ESCs (fold change > 2 and *p* < 0.05). Green and red values represent fold changes for down- and upregulation, respectively. **b** The numbers of up- and downregulated genes induced by *Cited1* overexpression in ESCs (fold change > 2 and *p* < 0.05). **c** qRT-PCR analysis to validate the expression changes of DEGs identified by the microarray assay in ESCs 48 h after *Cited1* overexpression. The average mRNA level in control cells transfected with an empty vector pPy was set at 1.0. Data are shown as mean ± SD (*n* = 3). *p < 0.05, **p < 0.01, ***p < 0.001. **d** Significantly enriched GO terms of the top 60 DEGs induced by *Cited1* overexpression at day 2 compared with those from empty vector pPy overexpression. **e** A venn diagram showing intersections of 3 sets of DEGs induced by *Cited1* overexpression (pink), *Gata3* overexpression (blue), or *Cdx2* overexpression (green), with gene numbers indicated. Out of 696 DEGs induced by *Cited1*, 462 DEGs were shared with those induced by *Gata3* or *Cdx2*. **f** The GSEA using ordered gene expression levels from *Cited1*-overexpressing cells over control ESCs (*X*-axis) with gene sets indicated. *Cdx2* OE (368 genes), and *Gata3* OE (384 genes) in ESCs, top 1% of upregulated genes upon *Oct4* KD (204 genes), TSC-specific (313 genes) and ESC-specific (218 genes) gene sets

To evaluate the ability of Cited1 to control ESC fate determination at a genome-wide scale, we analyzed the global transcriptional changes caused by *Cited1* overexpression in ESCs. Cells were collected at day 1 and day 2 after transfection and genome-wide transcript profiles were determined by Affymetrix microarray analysis (Fig. 5a). With a cutoff threshold of two-fold and *p* value < 0.05, 40 and 696 differentially expressed genes (DEGs) were identified at day 1 and day 2, respectively (Fig. 5b; Table S2). Twelve DEGs were randomly selected for qRT-PCR validation (Fig. 5c). Top 60 DEGs induced by *Cited1* overexpression was further analyzed by GO (Gene Ontology). GO terms related to embryonic organ development and embryonic placenta development were enriched (Fig. 5d), in accordance with the phenotype caused by *Cited1* overexpression in vitro and in vivo.

Several studies have reported that overexpression of key trophoblast genes (*Cdx2* or *Gata3*) could induce mouse ESCs to become trophoblast-like cells^9,10^. To know whether *Cited1* overexpression would induce similar transcriptional programs, we compared the global gene expression profiles of *Cited1*-overexpressing cells with previously published microarray data from *Cdx2*- and *Gata3-*overexpressing cells^10^. Interestingly, 60.1% (462 out of 696) of the DEGs induced by *Cited1* overexpression were overlapped with the DEGs induced by either *Cdx2* or *Gata3* (Fig. 5e), suggesting that *Cited1*-overexpressing cells gained similar molecular features to trophoblast-like cells induced by overexpressing *Cdx2* or *Gata3*.

We next performed gene set enrichment analysis (GSEA) to compare our microarray data of *Cited1*-overexpressing cells with the following gene sets: top 1% of upregulated *Cdx2* OE-specific genes (368 genes), *Gata3* OE-specific genes (384 genes) or *Oct4* KD-specific genes (204 genes) or TSC-specific genes (TSCs vs. ESCs, 313 genes), and ESC-specific genes (ESCs vs. differentiated ESCs, 218 genes)^10,12,44^. The analysis revealed that *Cited1* OE-specific DEGs were strongly positively correlated with the TSC-specific gene sets and the gene profiles of trophoblast-like cells generated by *Cdx2* OE or *Gata3* OE, or *Oct4* KD (Fig. 5f). In contrast, these *Cited1* OE-specific DEGs displayed a poor correlation with ESC-specific gene sets. Taken together, The analysis indicates that ectopic expression of *Cited1* in ESCs can result in the loss of ESC identity and evoke an overall startup of trophoblast-like gene expression program.

### Cited1 interacts with Bmpr2 to activate BMP signaling and induce trophoblast trans-differentiation

**Fig. 6:**
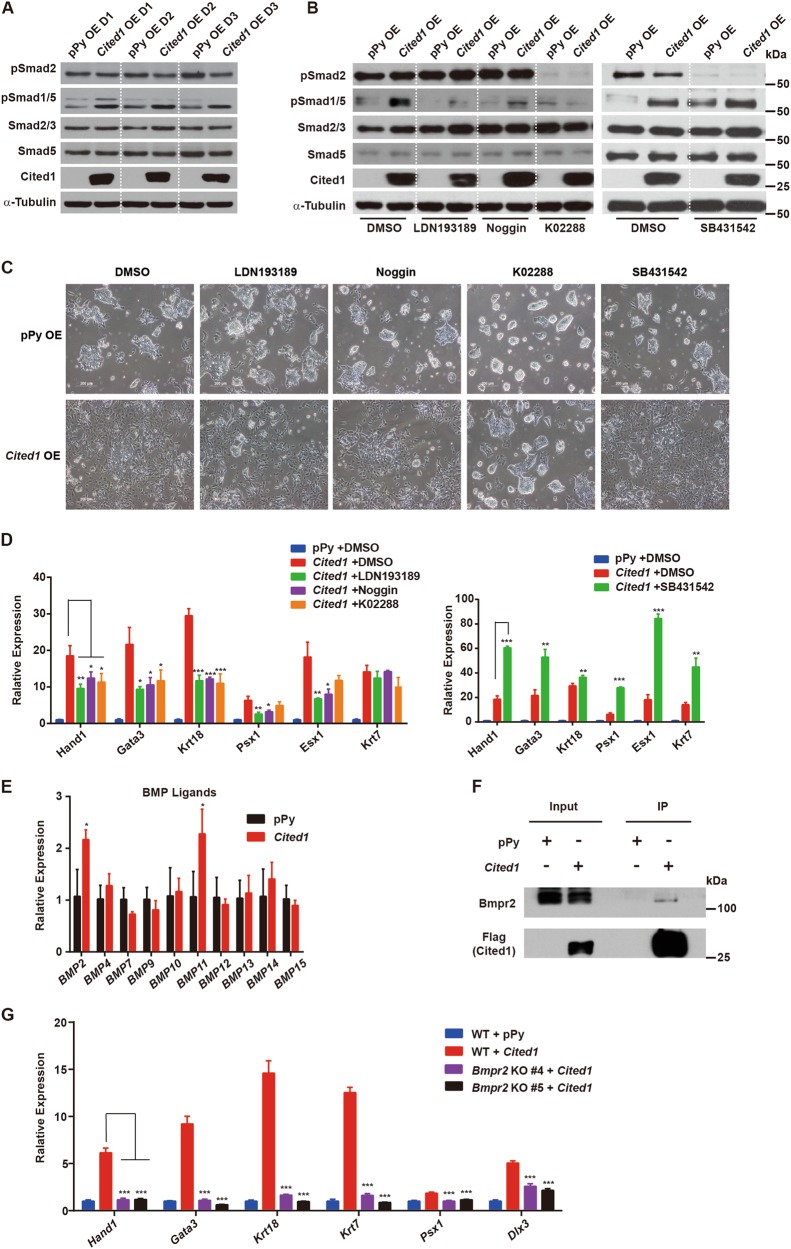
Cited1 interacts with Bmpr2 to activate BMP signaling and induce trophoblast differentiation. **a** Protein levels of pSmad1/5 and pSmad2 upon *Cited1* overexpression in E14T ESCs at the times as indicated. Smad5, Smad2/3, and α-Tubulin were used as loading controls. **b** Protein levels of pSmad1/5 and pSmad2 upon *Cited1* overexpression and inhibitor treatment for 3 days in E14T ESCs. Smad5, Smad2/3, and α-Tubulin were used as loading controls. **c** Bright field images of *Cited1*-overexpressing or control ESCs after treatment with DMSO or different inhibitors for 3 days. LDN193189 (0.1 μM), Noggin (400 ng/mL) and K02288 (10 μM) are all BMP signaling inhibitors. SB431542 (10 μM) is a TGF-β signaling inhibitor. **d** qRT-PCR analysis of transcript levels of trophoblast markers after transfection and treatment with inhibitors for 3 days. The average mRNA level in cells transfected with control vector pPy and treated with DMSO was set at 1.0. Data are shown as mean ± SD (*n* = 3). *p < 0.05, **p < 0.01, ***p < 0.001. **e** qRT-PCR analysis of mRNA levels for ligands of the BMP pathway induced by *Cited1* overexpression in E14T ESCs for 3 days. The average mRNA level in cells transfected with control vector pPy was set at 1.0. Data are shown as mean ± SD (*n* = 3). *p < 0.05. **f** The representative western blot result from Co-IP (immunoprecipitation) assays showing that ectopically expressed Flag-Cited1 binds to endogenous Bmpr2 specifically in E14T ESCs. Whole-cell lysates (WCL) were immunoprecipitated with anti-Flag M2 beads and analyzed by western blotting with antibodies as indicated. Five percent of WCL was loaded as the input. **g** qRT-PCR analysis of mRNA levels of trophoblast specific markers in parental WT ESCs or *Bmpr2* knockout (KO) cells upon *Cited1* overexpression for 3 days. The average mRNA level in WT cells transfected with empty vector pPy was set at 1.0. Data are shown as mean ± SD (*n* = 3). ***p *<* 0.001

We were interested in how Cited1 activated trophoblast-like transcriptional program. Cited1 was reported to bind nuclear Smads to enhance their transcriptional activity^33,34^ and hyperactivation of BMP4-Smad1/5/8 signaling was known to induce trophoblast differentiation in ESCs^19,21^. Therefore, we examined whether the phenotype induced by *Cited1* overexpression would be associated with the activation of BMP-Smad1/5/8 signaling. We first evaluated levels of phosphorylated Smad1/5 (pSmad1/5) and Smad2 (pSmad2) in *Cited1*-overexpressing cells. Levels of pSmad1/5 were dramatically elevated 1 day after *Cited1* overexpression (Fig. 6a), indicating that Cited1 activated BMP-Smad1/5 signaling. Meanwhile, the levels of pSmad2 slightly decreased, hinting at a potential involvement of Cited1 in the regulation of Nodal/Activin signaling. To verify whether the activation of BMP-Smad1/5 signaling was responsible for Cited1-induced trophoblast-like trans-differentiation, BMP signaling inhibitors, including Noggin (a ligand antagonist), LDN193189 and K02288 (inhibitors targeting type I receptors), were respectively added to the culture media 6 h after *Cited1* transfection. SB431542, a TGF-β signaling inhibitor, was used as a negative control. The effectiveness of inhibitors was verified by western blotting (Fig. 6b). Results from both cell morphology and marker gene expression analysis at day 3 indicated that Noggin or K02288, or LDN193189 treatment significantly compromised trophoblast trans-differentiation induced by *Cited1* overexpression (Fig. 6c, d, left panel). In contrast, SB431542 enhanced the expression of trophoblast markers induced by *Cited1* overexpression (Fig. 6d, right panel), which was in line with the reports that SB431542 promotes human ESC differentiation into trophoblast^24^ and that Nodal/activin signaling regulates BMP signaling in mouse ESCs^45,46^. The results were reproducible in another mouse ESC line (Figure S6). Therefore, activation of BMP signaling might play a major role for Cited1 to induce trophoblast-like trans-differentiation.

To explore how Cited1 activated BMP signaling, we determined the transcript levels of BMP signaling ligands in *Cited1*-overexpressing cells at day 3 and found that most ligands tested had comparable levels to those in control cells (Fig. 6e). Since ectopic Cited1 mainly located in the cytoplasm (Figure S7A), we examined if Cited1 could bind BMP receptors. Co-immunoprecipitation (Co-IP) assays showed that ectopic Cited1 interacted with endogenous Bmpr2 in ESCs (Fig. 6f), suggesting Cited1 might activate BMP-Smad1/5 signaling via its interaction with Bmpr2. To determine the role of Bmpr2 for Cited1’s function, two *Bmpr*2 KO ESC lines were generated by the CRISPR/Cas9 system (Figure S7B). *Cited1* overexpression could not activate trophoblast marker expression in both lines of *Bmpr*2 KO ESCs (Fig. 6g), indicating that Bmpr2 might be a major mediator of Cited1 to activate trophoblast-associated gene expression, although it is not clear how Cited1 modulates the function of Bmpr2.

### Cited1 links to Smad4 and p300 to enhance the transcriptional activity of BMP4 signaling effectors

**Fig. 7:**
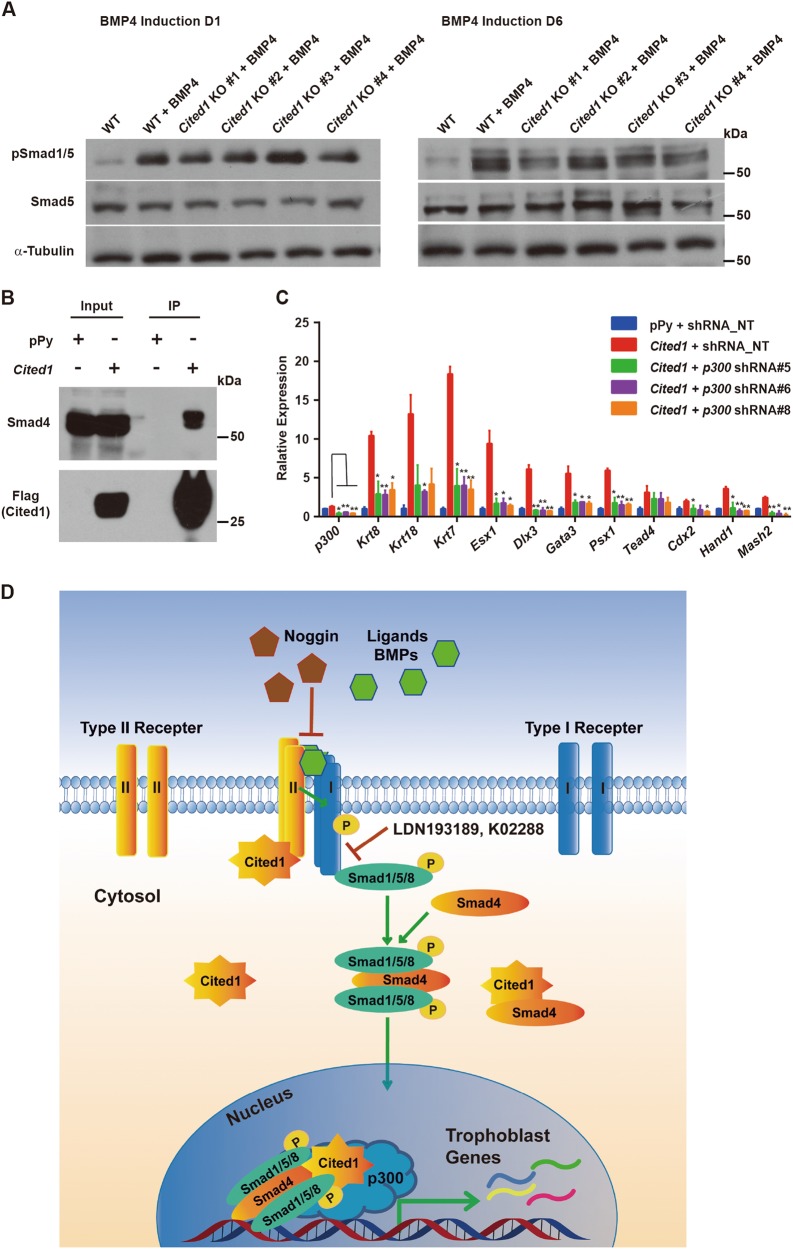
Cited1 binds to Smad4 and its induction of trophoblast trans-differentiation depends on *p300*. **a** Protein levels of pSmad1/5 in WT ESCs and four *Cited1* KO ESC clones after treatment with BMP4 for 1 day and 6 days. **b** Co-IP assay to show that exogenously expressed Flag-Cited1 binds to endogenous Smad4 specifically in E14T ESCs. Whole-cell lysates (WCL) were immunoprecipitated with anti-Flag M2 beads and analyzed by western blotting with anti-Smad4 and anti-Flag antibodies, respectively. Five percent of the WCL was loaded as the input. **c** qRT-PCR analysis of transcript levels of trophoblast specific markers in control E14T ESCs (shRNA_ NT) or *p300* KD cells (*p300* shRNA#5, 6, 8) upon *Cited1* overexpression for 3 days. The average mRNA level in cells transfected with an empty vector (pPy) and a non-targeting shRNA plasmid (shRNA_NT) was set at 1.0. Data are shown as mean ± SD (*n* = 3). *p < 0.05, **p *<* 0.01. **d** A proposed working model for Cited1 to promote mouse ESC trans-differentiation toward a trophoblast-like state through activation of BMP signaling. On the one hand, Cited1 binds to Bmpr2 to enhance the phosphorylation of Smad1/5/8. On the other hand, Cited1 may functionally link Smad4 and p300 to activate trophoblast trans-differentiation associated transcriptional program

The observations that the level of pSmad1/5 was only moderately reduced in *Cited1* KO cells after BMP4 treatment (Fig. 7a) and that a small proportion of Cited1 located in the nucleus (Figure S7A) prompted us to consider whether Cited1 would have a nuclear function in addition to its interaction with Bmpr2. As Cited1 was previously reported to bind Smad4 and could form complexes with Smad1/5/8 and p300/Cbp to enhance the activity of BMP signaling^34,47^, we speculated that Cited1 might also positively regulate BMP4 signaling through associating with Smads. To test the possibility, Co-IP assays were performed in ESCs. Indeed, Flag-Cited1 and endogenous Smad4 formed complexes (Fig. 7b). As Cited1 is a coactivator of the p300/Cbp-mediated transcription, we further tested whether the induction of trophoblast genes by *Cited1* overexpression was dependent on p300. Expression of *p300* was silenced by three sets of shRNAs specifically targeting different coding sequences (shRNA#5, #6, #8), respectively. KD of *p300* abolished the activation of trophoblast genes induced by *Cited1* overexpression (Fig. 7c), revealing the dependence of Cited1 on p300 for induction of trophoblast gene expression. Taken together, we propose that Cited1 induces a trophoblast transcriptional signature in ESCs through interacting with Bmpr2, Smad4, and p300 to activate BMP signaling (Fig. 7d).

## Discussion

In this study, we report that transcription coactivator Cited1 can induce mouse ESCs into a trophoblast-like state through activation of the BMP signaling pathway. Interestingly, after *Cited1* overexpression, trophoblast-related genes were massively upregulated whereas pluripotency genes were only slightly downregulated, indicating that ectopic *Cited1* could directly activate a trophoblast-like transcriptional signature. The downregulation of pluripotency genes could be a secondary event.

Although some markers (such as *Gata3*, *Hand1*, and *Cdx2*) induced by Cited1 have been implicated in both mesendoderm and trophoblast differentiation, our results showed that Cited1 activated trophoblast genes more robustly than mesendoderm sole genes (Fig. 3b, e and S4F). Moreover, *Cited1* overexpression did not induce expression of mesendoderm master gene T (Figure S4G). Combined with the phenotype of typical trophoblastic tumors generated from *Cited1*-overexpressing cells (Fig. 4) and a genome-wide trophoblast-like gene expression program evoked by ectopic *Cited1* (Fig. 5), we conclude that Cited1 induces trophoblast-like differentiation from ESCs under a self-renewal condition. However, we do not exclude the possibility that Cited1 might also function in the mesendoderm development under certain contexts.

BMP4 is an import signal to trigger trophoblast differentiation. However, the intracellular factors that can directly activate the BMP pathway remain to be discovered. Our study uncovers that Cited1 associates with and functionally depends on Bmpr2 to activate Smad1/5, the effectors of BMP signaling. Furthermore, our results reveal that Cited1 forms protein complexes with Smad4 and requires p300 to activate trophoblast gene expression, in a similar way to its activation of the TGF-β or BMP pathway in other cell contexts reported previously^33,34,36,47^. Taken together, this study shows that Cited1 directly activates the BMP pathway to induce the conversion of ESCs into trophoblast-like cells via two complementary mechanisms in the cytoplasm and nucleus, respectively.

## Experimental procedures

### Cell culture

Mouse E14T, CGR8, and ZHBTc4 ESCs (kind gifts of Dr. Austin Smith from the University of Cambridge) were cultured on gelatin-coated plates in the ESC medium consisting of GMEM (Glasgow’s minimum essential medium; Invitrogen, cat 11710–035) supplemented with 10% fetal bovine serum (FBS; Gibco), 1 mM sodium pyruvate (Gibco), 0.1 mM non-essential amino acids (NEAA; Gibco), 2 mM l-glutamine (Gibco), 100 μM β-mercaptoethanol (Sigma), 100 U/mL penicillin and 100 μg/mL streptomycin (Hyclone), and 1000 U/mL recombinant leukemia inhibitory factor (LIF; Millipore). Mouse TSCs (a kind gift of Dr. Shaorong Gao from Tongji University) were cultured as described previously^21^.

### Trophoblast Trans-differentiation Induced by BMP4

For trophoblast induction by BMP4, mouse ESCs were plated in Matrigel-coated six-well plates at a density of 1.0 × 10^5^ cells per well in a trophoblast induction medium for 6 days. The trophoblast induction medium contains KnockOut DMEM/F-12 (Invitrogen, cat 12660–012), 64 μg/mL l-ascorbic acid-2-phosphate magnesium (ACC), insulin-transferrin-selenium (Life Technologies), 543 μg/mL NaHCO_3_ (Sigma), 1 μg/mL heparin (Sigma), 2 mM l-glutamine (Life Technologies), 100 U/mL penicillin and 100 μg/mL streptomycin (Hyclone), and 10 ng/mL BMP4 (HUMANZYME).

### Inhibitors and antibodies used in western blot analysis

Western blotting was carried out with following primary antibodies: Cited1 (made in our lab or Genetex, GTX114559), Oct4 (made in our lab), Sox2 (made in our lab), phospho-Smad1/5 (Cell Signaling Technology, #9516), phospho-Smad2 (Cell Signaling Technology, #3101), total Smad5 (Cell Signaling Technology, #9517), total Smad2/3 (Cell Signaling Technology, #3102), Smad4 (Proteintech, 10231–1-AP), Bmpr2 (Abcam, ab96826), Elf5 (Santa Cruz, sc-9645), T (R&D, AF2085), Placental lactogen 1 (Santa Cruz, sc-376436), Flag (Abmart, M20018F), β-Actin (HuaBio, M1210–2) and α-Tubulin (Sigma, T5168). The inhibitors were purchased from STEMCELL (SB431542: #72232), Selleckchem (LDN193189: S2618; K02288: S7359; protease inhibitor cocktail: B14002; phosphatases inhibitor: B15002) and PeproTech (Noggin: #120–10 C), respectively.

### Mouse embryo collection and immunofluorescence analysis

Mouse embryo collection and immunofluorescence were carried out as described previously^8,41^. Briefly, pre-implantation embryos were flushed out from the oviducts or uterus of pregnant female mice and were subsequently fixed in 4% paraformaldehyde. Immunofluorescence staining was performed using primary antibodies against Cdx2 (Biogenex, MU392A-UC), Oct4 (Santa Cruz, sc-101534) or Cited1 (Genetex, GTX114559), following standard protocols for whole embryo staining^8^. Image data were acquired with a Zeiss LSM 780 microscope^41^. The cell immunofluorescence staining was performed with following steps: ESCs on coverslips were firstly fixed with 4% paraformaldehyde for 15 min, permeabilized with 0.2 % Triton X-100 for 10 min, and blocked with 3% bovine serum albumin (BSA) for 30 min. Next, cells were incubated with the primary anti-Cdx2 antibody (1:200 diluted), or anti-Elf5 antibody (1:200 diluted), or anti-Krt7 antibody (1:200 diluted), overnight at 4 °C. The next day, cells were incubated with the fluorescent Cy3-goat anti-mouse antibody (1:200 diluted), or FITC-goat anti-mouse antibody (1:200 diluted), or Cy3-rabbit anti-goat antibody (1:200 diluted), for 1 h at room temperature in the dark, respectively. The nuclei were further stained with 4′,6-diamidino-2-phenylindole (DAPI; Invitrogen) for 3 min. Finally, coverslips were dried and affixed to slides using a fluorescent mounting medium^21^.

### Immunohistochemistry

Immunofluorescence was performed as described by the laboratory of Dr. Janet Rossant (http://lab.research.sickkids.ca/rossant/labresources/). An antibody was used against Placental lactogen 1 (sc-376436; 1:50 dilution).

### Mesoderm induction

For mesoderm induction by BMP4 and CHIR99021, mouse ESCs were plated in gelatin-coated six-well plates at a density of 1.5 × 10^5^ cells per well in a mesoderm induction medium for 3 days. The mesoderm induction medium contains N2B27 medium supplemented with 1% knock-out serum replacement (KOSR, Gibco), 0.1% bovine serum albumin (Gibco) and BMP4 at 10 ng/mL (HUMANZYME) for 2 days. Cells were then changed to a DMEM-based medium, supplemented with 15% KOSR, 0.5% DMSO (Sigma) and CHIR99021 (Stem cell) at 1 µM^48^.

### Flow cytometry analysis

Cell cultures were dispersed as single-cell suspensions by 0.25% trypsin containing EDTA (Invitrogen). After fixation in a 4% paraformaldehyde/PBS solution for 15 min and permeabilization in 1.0% Triton X-100/PBS for 15 min, nonspecific binding was reduced by exposure to 5% donkey serum in 0.2% Triton X-100/PBS for 30 min at room temperature. The cells were incubated with primary anti-Krt7 (Santa Cruz, sc-53263) antibody (1:25 diluted) for 1 h at room temperature, followed by incubation with FITC-donkey anti-mouse antibody (1:200 diluted) for 30 min at room temperature. Between all steps, cells were washed three times with a PBS buffer. All procedures were carried out in the dark at room temperature. Cells were analyzed on a Beckman Coulter Gallios flow cytometer system. Final data were prepared in FlowJo (version 10) software. All flow cytometry analyses were carried out three times and representative results are shown.

### Plasmids and transfection

The coding sequences of full-length *Cited1* [NCBI Reference Sequence: NM_001276474.1] and its truncations were amplified using Hi-Fi KOD polymerase (Takara) from complementary DNA (cDNA) of E14T mouse ESCs. Amplified fragments were subcloned into the pPyCAGIP vector (a kind gift of Dr. Ian Chambers from University of Edinburgh). Three short-hairpin RNAs (shRNAs) targeting *p300* mRNA were synthesized (by Shanghai Shengong) and subcloned into pLKO.1-puro (Addgene) vector^49^. Primers used for gene cloning and shRNA targeting sequences are listed in Table S3. For transfection, ESCs were plated in six-well plates at a density of 0.2 million cells per well 1 day before transfection. The transfection was performed using Lipofectamine™2000 (Invitrogen) mixed with appropriate plasmids according to the manufacturer’s instruction.

### RNA isolation, reverse transcription and quantitative real-time PCR (qRT-PCR)

Total RNA was isolated using the TRIzol Reagent (Invitrogen) and was reverse-transcribed to cDNA by the ReverTra Ace (Toyobo, TRT-101). The real-time qPCR was performed using the SYBR® *Premix Ex Taq*™ II (Takara) mixed with appropriate primers on a ViiA 7 real-time PCR system (Life Technologies). *Gapdh* was used as an internal control to normalize the relative expression level of each gene.

### Teratomas

For *Cited1* experiment, ESCs were transfected with the *Cited1* expression plasmid or an empty pPyCAGIP vector. One day later, the cells were selected with 1 μg/mL puromycin (Sigma) for additional 2 days and then harvested. Subsequently, 1.5 million transfected cells were subcutaneously injected into NOD/SCID mice. Teratomas were collected 4 weeks later for histological analysis. For trophoblast induction experiment, ESCs were cultured in a trophoblast induction medium for 6 days to transdifferentiate into trophoblast-like cells. Then 1.5 million cells were subcutaneous injected into NOD/SCID mice. Teratomas were collected 6 weeks later for histological analysis.

### Western blot analysis and immunoprecipitation (IP)

Collected cells were washed twice with PBS and lysed in the Co-IP buffer (50 mM Tris-HCl (pH 7.5), 150 mM NaCl, 10% glycerol, 2 mM EDTA, 0.5% NP-40, and protease inhibitors). Protein concentrations were determined using the Pierce™ BCA Protein Assay Kit (Thermo Fisher) according to the manufacturer’s instructions. Total proteins were separated by SDS-PAGE gels and transferred to nitrocellulose membranes (GE Healthcare). Membranes were incubated with specific primary antibodies, and the antibody-protein complexes were detected by HRP-conjugated secondary antibodies (Jackson) and Pierce ECL Western Blotting Substrate (Thermo Fisher). All western blot analyses were carried out at least three times and representative results are shown. The IP assays were conducted as previously described^50^. Anti-Flag M2 magnetic beads (Sigma, M8823) were added to the cell lysate for incubation overnight.

### Alkaline phosphatase (AKP) staining assays

AKP staining assays were performed according to manufacturer’s instructions (VECTOR Blue Alkaline Phosphatase Substrate Kit, SK-5300, Vector Lab).

### Genome editing with CRISPR-Cas9 approach

The sgRNAs targeting *Cited1* and *Bmpr2* locus were designed using the CRISPR Design Tool (http://crispr.mit.edu/) and synthesized by the Shanghai Shenggong Company. Annealed sgRNA oligos were inserted into the pSpCas9(BB)-2A-Puro (PX459) (Addgene Plasmid #48139). E14T ESCs were plated onto gelatin-coated six-well plates to grow for 1 day, and then were transfected with sgRNA-PX459 plasmids targeting *Cited1* and *Bmpr2* using Lipofectamine™ 2000, respectively. One day after transfection, cells were selected by 1.5 μg/mL puromycin for 2 days. Then puromycin-resistant ESCs were replated at the density of 10 thousand cells per 10-cm dish and cultured for 6 days without selection. Colonies were picked up and verified by PCR and Sanger sequencing individually. The sgRNA sequences targeting *Cited1* or *Bmpr2* are included in Table S3.

### Microarray analysis

E14T ESCs were transiently transfected with a *Cited1* expression construct or an empty pPyCAGIP vector. One day later, transfected cells were selected in 1 μg/mL puromycin. Independent biological triplicates of either *Cited1* construct-transfected cells or control construct-transfected cells at day 1 and day 2 were collected for RNA isolation with Trizol. Microarray analysis was carried out with the Affymetrix GeneChip® Mouse Genome 430 2.0 Arrays. The whole procedures, including RNA extraction, cDNA synthesis, labeling, hybridization, washing and scanning, were conducted according to the standard Affymetrix protocol by the Shanghai Biotechnology Corporation. Differentially expressed genes (DEGs) with a fold change > 2 were selected for further analysis. The selected genes were grouped in functional categories based on Gene Ontology database (GO: http://www.geneontology.org/), and gene set enrichment analysis (GSEA) were also made^12^. The microarray data from this publication have been submitted to the GEO database with an accession number of GSE103414.

### Statistical analysis

All qRT-PCR data are presented as the mean ± SD of three independent experiments and analyzed by the unpaired Student’s *t*-test. *p* < 0.05 was considered statistically significant. *p < 0.05, **p < 0.01, ***p < 0.001.

## Electronic supplementary material

Supplemental figure legends

Figure S1. Related to Figure 1. BMP4 induces mouse ESC differentiation with trophoblast genes up-regulation

Figure S2. Related to Figure 1. Cited1 is upregulated during TSC differentiation and Cited1 mainly expressed in the plasm of cells in early embryos

Figure S3. Related to Figure 2. Genomic DNA sequences before and after the gRNA-mediated cleavage and repair in Cited1 loci

Figure S4. Related to Figure 3. Forced expression of Cited1 promotes mouse ESC differentiation into trophoblast-like cells

Figure S5. Related to Figure 3. Ectopic Cited1 induces the expression of trophoblast markers under LIF withdrawal condition and the function of Cited1 depends on its full-length

Figure S6. Related to Figure 6. Inhibition of BMP signaling pathway partially rescues the differentiation phenotype caused by Cited1 overexpression in CGR8 cells

Figure S7. Related to Figure 6. Genomic DNA sequences before and after the gRNA-mediated cleavage and repair in Bmpr2 loci

Table S1 Gene sets of TSC, transcription factor (TF), and Oct4 knockdown (KD) cells

Table S2 Differentially expressed genes (DEGs) in Cited1 OE, Cdx2 OE and Gata3 OE

Table S3 Sequences of primers for gene cloning, qRT-PCR and sgRNAs or shRNAs for gene targeting

## References

[1] Johnson MH, Ziomek CA (1981). The foundation of two distinct cell lineages within the mouse morula. Cell.

[2] Morris SA (2010). Origin and formation of the first two distinct cell types of the inner cell mass in the mouse embryo. Proc. Natl Acad. Sci. USA.

[3] Rossant J, Tam PP (2009). Blastocyst lineage formation, early embryonic asymmetries and axis patterning in the mouse. Development.

[4] Evans MJ, Kaufman MH (1981). Establishment in culture of pluripotential cells from mouse embryos. Nature.

[5] Martin GR (1981). Isolation of a pluripotent cell line from early mouse embryos cultured in medium conditioned by teratocarcinoma stem cells. Proc. Natl Acad. Sci. USA.

[6] Tanaka S, Kunath T, Hadjantonakis AK, Nagy A, Rossant J (1998). Promotion of trophoblast stem cell proliferation by FGF4. Science.

[7] Beddington RS, Robertson EJ (1989). An assessment of the developmental potential of embryonic stem cells in the midgestation mouse embryo. Development.

[8] Strumpf D (2005). Cdx2 is required for correct cell fate specification and differentiation of trophectoderm in the mouse blastocyst. Development.

[9] Tolkunova E (2006). The caudal-related protein cdx2 promotes trophoblast differentiation of mouse embryonic stem cells. Stem Cells.

[10] Ralston A (2010). Gata3 regulates trophoblast development downstream of Tead4 and in parallel to Cdx2. Development.

[11] Rhee C (2017). ARID3A is required for mammalian placenta development. Dev. Biol..

[12] Rhee C (2014). Arid3a is essential to execution of the first cell fate decision via direct embryonic and extraembryonic transcriptional regulation. Genes Dev..

[13] Vong QP (2010). A role for borg5 during trophectoderm differentiation. Stem Cells.

[14] Nichols J (1998). Formation of pluripotent stem cells in the mammalian embryo depends on the POU transcription factor Oct4. Cell.

[15] Niwa H, Miyazaki J, Smith AG (2000). Quantitative expression of Oct-3/4 defines differentiation, dedifferentiation or self-renewal of ES cells. Nat. Genet..

[16] Niwa, H. et al. Interaction between Oct3/4 and Cdx2 determines trophectoderm differentiation. *Cell* **123**, 917–929 (2005). 10.1016/j.cell.2005.08.040 16325584

[17] Koh, K. P. et al. Tet1 and Tet2 regulate 5-hydroxymethylcytosine production and cell lineage specification in mouse embryonic stem cells. *Cell Stem Cell* **8**, 200–213 (2011). 10.1016/j.stem.2011.01.008 PMC3134318 21295276

[18] Xu RH (2002). BMP4 initiates human embryonic stem cell differentiation to trophoblast. Nat. Biotechnol..

[19] Hayashi Y (2010). BMP4 induction of trophoblast from mouse embryonic stem cells in defined culture conditions on laminin. In. Vitr. Cell. Dev. Biol. Anim..

[20] Li Y (2013). BMP4-directed trophoblast differentiation of human embryonic stem cells is mediated through a DeltaNp63+ cytotrophoblast stem cell state. Development.

[21] Liu J (2016). Single-stranded DNA binding protein Ssbp3 induces differentiation of mouse embryonic stem cells into trophoblast-like cells. Stem Cell Res. Ther..

[22] Das, P. et al. Effects of fgf2 and oxygen in the bmp4-driven differentiation of trophoblast from human embryonic stem cells. *Stem Cell Res.* **1**, 61–74 (2007). 10.1016/j.scr.2007.09.004 PMC2634289 19194525

[23] Schulz LC (2008). Human embryonic stem cells as models for trophoblast differentiation. Placenta.

[24] Wu Z (2008). Combinatorial signals of activin/nodal and bone morphogenic protein regulate the early lineage segregation of human embryonic stem cells. J. Biol. Chem..

[25] Lin G, Martins-Taylor K, Xu RH (2010). Human embryonic stem cell derivation, maintenance, and differentiation to trophoblast. Methods Mol. Biol..

[26] Erb TM (2011). Paracrine and epigenetic control of trophectoderm differentiation from human embryonic stem cells: the role of bone morphogenic protein 4 and histone deacetylases. Stem Cells Dev..

[27] Marchand M (2011). Transcriptomic signature of trophoblast differentiation in a human embryonic stem cell model. Biol. Reprod..

[28] Amita M (2013). Complete and unidirectional conversion of human embryonic stem cells to trophoblast by BMP4. Proc. Natl Acad. Sci. USA.

[29] Koel, M. et al. Optimizing bone morphogenic protein 4-mediated human embryonic stem cell differentiation into trophoblast-like cells using fibroblast growth factor 2 and transforming growth factor-beta/activin/nodal signalling inhibition. *Reprod. Biomed. Online* **35**, 253–263 (2017). 10.1016/j.rbmo.2017.06.003 28647356

[30] ten Dijke, P. & Hill, C. S. New insights into TGF-beta-Smad signalling. *Trends Biochem. Sci.* **29**, 265–273 (2004). 10.1016/j.tibs.2004.03.008 15130563

[31] Record, M. Intercellular communication by exosomes in placenta: a possible role in cell fusion? *Placenta* **35**, 297–302 (2014). 10.1016/j.placenta.2014.02.009 24661568

[32] Shioda T, Fenner MH, Isselbacher KJ (1996). msg1, a novel melanocyte-specific gene, encodes a nuclear protein and is associated with pigmentation. Proc. Natl Acad. Sci. USA.

[33] Shioda T (1998). Transcriptional activating activity of Smad4: roles of SMAD hetero-oligomerization and enhancement by an associating transactivator. Proc. Natl Acad. Sci. USA.

[34] Plisov S (2005). Cited1 is a bifunctional transcriptional cofactor that regulates early nephronic patterning. J. Am. Soc. Nephrol..

[35] Yahata T (2001). Selective coactivation of estrogen-dependent transcription by CITED1 CBP/p300-binding protein. Genes Dev..

[36] Cantelli, G. et al. TGF-beta-induced transcription sustains amoeboid melanoma migration and dissemination. *Curr. Biol.* **25**, 2899–2914 (2015). 10.1016/j.cub.2015.09.054 PMC4651903 26526369

[37] Ahmed NU, Shioda T, Coser KR, Ichihashi M, Ueda M (2001). Aberrant expression of MSG1 transcriptional activator in human malignant melanoma in vivo. Pigment Cell Res..

[38] Meniel V (2013). Cited1 deficiency suppresses intestinal tumorigenesis. PLoS Genet..

[39] Lovvorn HN (2007). Wilms’ tumorigenesis is altered by misexpression of the transcriptional co-activator, CITED1. J. Pediatr. Surg..

[40] Rodriguez TA (2004). Cited1 is required in trophoblasts for placental development and for embryo growth and survival. Mol. Cell. Biol..

[41] Zheng XF (2016). CNOT3-dependent mRNA deadenylation safeguards the pluripotent state. Stem Cell Rep..

[42] Ran, F. A. et al. Genome engineering using the CRISPR-Cas9 system. *Nat. Protoc.* **8**, 2281–2308 (2013). 10.1038/nprot.2013.143 PMC3969860 24157548

[43] Shioda T, Fenner MH, Isselbacher KJ (1997). MSG1 and its related protein MRG1 share a transcription activating domain. Gene.

[44] Hailesellasse Sene K (2007). Gene function in early mouse embryonic stem cell differentiation. BMC Genom..

[45] Galvin KE, Travis ED, Yee D, Magnuson T, Vivian JL (2010). Nodal signaling regulates the bone morphogenic protein pluripotency pathway in mouse embryonic stem cells. J. Biol. Chem..

[46] Guzman-Ayala, M. et al. Graded Smad2/3 activation is converted directly into levels of target gene expression in embryonic stem cells. *PLoS ONE* **4**, doi:ARTN e426810.1371/journal.pone.0004268 (2009). 10.1371/journal.pone.0004268 PMC2627943 19172185

[47] Yahata T (2000). The MSG1 non-DNA-binding transactivator binds to the p300/CBP coactivators, enhancing their functional link to the Smad transcription factors. J. Biol. Chem..

[48] Chal J (2015). Differentiation of pluripotent stem cells to muscle fiber to model Duchenne muscular dystrophy. Nat. Biotechnol..

[49] Stewart SA (2003). Lentivirus-delivered stable gene silencing by RNAi in primary cells. RNA.

[50] Xu HM (2004). Wwp2, an E3 ubiquitin ligase that targets transcription factor Oct-4 for ubiquitination. J. Biol. Chem..

