# Supplementary material for: Transcription coactivator Cited1 acts as an inducer of trophoblast-like state from mouse embryonic stem cells through the activation of BMP signaling
Source: Cell Death Dis. 2018 Sep 11;9(9):924. doi: 10.1038/s41419-018-0991-1 (PMC6134011; doi:10.1038/s41419-018-0991-1)
Supplement: Supplementary file 1 — Supplemental figure legends [file 41419_2018_991_MOESM1_ESM.docx]

**Supplemental figure legends**

**Figure S1. Related to Figure 1. BMP4 induces mouse ESC differentiation with trophoblast genes up-regulation**

(A) Immunofluorescence staining of ESCs exposed to BMP4 for 6 days. Samples were stained with anti-Oct4 (red) and anti-Krt7 (red) antibodies respectively, and DAPI staining highlighted the nuclei (blue). Scale bar: 50 μm.

(B) Flow cytometry density plots for Krt7 expression in ESCs after BMP4 induction for 6 days. Cells were fully dispersed, fixed, and immunostained for Krt7. For the negative control (NC) cells were exposed only to secondary antibody without prior exposure to primary Krt7.

(C) The statistical analysis of the flow cytometry data for Krt7 expression in ESCs exposed to BMP4 for 6 days. Data are shown as mean ± SD (n = 3). ****p* < 0.001.

(D) Cross-section of H & E staining for teratomas derived from control cells or BMP4-treated ESCs. Teratomas derived from BMP4-treated ESCs contained numerous hemorrhagic loci, which were indicated by arrowheads. The trophoblast giant cells with the enlarged nuclei (arrows) were indicated. Scale bar: 100 μm (left panel), 20 μm (right panel).

**Figure S2.** **Related to Figure 1. *Cited1* is upregulated during TSC differentiation and Cited1 mainly expressed in the plasm of cells in early embryos**

(A) Expression levels of *Cited1* and trophoblast marker genes over a TSC differentiation time course examined by qRT-PCR. The average mRNA level in TSCs was set at 1.0. Data are shown as mean ± SD (n = 3). **p* < 0.05, ***p* < 0.01, ****p* < 0.001.

(B) Immunostaining of Cited1 in early mouse embryos. Immunostaining of CNOT3 was used to indicate the cytosol. Scale bar: 20 μm.

**Figure S3. Related to Figure 2. Genomic DNA sequences before and after the gRNA-mediated cleavage and repair in *Cited1* loci**

(A) Sanger sequencing data of *Cited1* homogenous knockout lines #1, #2, #3 and #4 confirmed the deletion and shift-frame of *Cited1* loci in these clones. The Sanger sequencing peak maps also verified the precise rejoining of the double blunt ends.

**Figure S4. Related to Figure 3. Forced expression of Cited1 promotes mouse ESC differentiation into trophoblast-like cells**

(A) Cited1 protein levels in E14T ESCs transfected with control empty vector pPy, or *Cited1-*expressing plasmids were examined by western blot analysis. One day after transfection, ESCs were selected by puromycin for additional 1 day (overexpression for 2 days, OE D2).

(B) [The](http://cn.bing.com/dict/clientsearch?mkt=zh-CN&setLang=zh&form=BDVEHC&ClientVer=BDDTV3.5.0.4311&q=%E7%BB%86%E8%83%9E%E5%A2%9E%E6%AE%96%E6%9B%B2%E7%BA%BF) [cell](http://cn.bing.com/dict/clientsearch?mkt=zh-CN&setLang=zh&form=BDVEHC&ClientVer=BDDTV3.5.0.4311&q=%E7%BB%86%E8%83%9E%E5%A2%9E%E6%AE%96%E6%9B%B2%E7%BA%BF) [growth](http://cn.bing.com/dict/clientsearch?mkt=zh-CN&setLang=zh&form=BDVEHC&ClientVer=BDDTV3.5.0.4311&q=%E7%BB%86%E8%83%9E%E5%A2%9E%E6%AE%96%E6%9B%B2%E7%BA%BF) [curve](http://cn.bing.com/dict/clientsearch?mkt=zh-CN&setLang=zh&form=BDVEHC&ClientVer=BDDTV3.5.0.4311&q=%E7%BB%86%E8%83%9E%E5%A2%9E%E6%AE%96%E6%9B%B2%E7%BA%BF) of E14T cells transfected with pPy, or *Cited1* plasmids. Data are shown as mean ± SD (n = 3). **p* < 0.05, ***p* < 0.01, ****p* < 0.001.

(C) Morphology changes of CGR8 cells overexpressing *Cited1* for 2 days.

(D-E) Expression levels of pluripotency markers (D), three germ layer markers and trophoblast markers (E) in CGR8 ESCs expressing *Cited1* for 3 days were determined by qRT-PCR analyses. The average mRNA level in cells transfected with the empty vector was set at 1.0. Data are shown as mean ± SD (n = 3). **p* < 0.05, ***p* < 0.01, ****p* < 0.001.

(F) qRT-PCR analysis of the expression levels of pluripotency and three germ layer markers after transfection of plasmids as indicated in ESCs over a time course. The average mRNA level in cells transfected with the control vector pPy was set at 1.0. Data are shown as mean ± SD (n = 3). **p* < 0.05, ***p* < 0.01, ****p* < 0.001.

(G) Immunofluorescence staining of ESCs after transfection of *Cited1* for 6 days. Samples were stained with anti-T antibody (red). E14T ESCs were stained as a negative control and mesodermal cells induced from ESCs were stained as a positive control. DAPI staining highlighted the nuclei (blue). Scale bar: 50 μm.

**Figure S5. Related to Figure 3. Ectopic Cited1 induces the expression of trophoblast markers under LIF withdrawal condition and the function of Cited1 depends on its full-length**

(A-E) Expression levels of pluripotency markers (A), ectoderm markers (B), mesoderm markers (C), primitive endoderm and endoderm markers (D), and trophoblast markers (E) in E14T cells overexpressing *Cited1* for indicated time under LIF withdrawal condition were determined by qRT-PCR analyses. The average mRNA level in cells cultured in self-renewal condition transfected with the empty vector was set at 1.0. Data are shown as mean ± SD (n = 3). **p* < 0.05, ***p* < 0.01, ****p* < 0.001.

(F) The schematic diagrams of the Cited1 full-length protein sequence and various truncation mutants. SID: Smad Interaction Domain, CR1: Conserved Region 1, CR2: Conserved Region 2.

(G) The typical morphology of cells expressing *Cited1* and its truncation mutants for 2 days.

(H) qRT-PCR analysis for the expression levels of trophoblast markers in ESCs 2 days after transfection of plasmids as indicated. The average mRNA level in cells transfected with the control vector pPy was set at 1.0. Data are shown as mean ± SD (n = 3). **p* < 0.05, ***p* < 0.01.

**Figure S6. Related to Figure 6. Inhibition of BMP signaling pathway partially rescues the differentiation phenotype caused by Cited1 overexpression in CGR8 cells**

(A) Protein levels of pSmad1/5 and pSmad2 upon *Cited1* overexpression in CGR8 ESCs over a time course. Smad5, Smad2/3 and α-Tubulin were used as loading controls.

(B) Protein levels of pSmad1/5 and pSmad2 upon *Cited1* overexpression and inhibitor treatment for 3 days in CGR8 cells. Smad5, Smad2/3 and α-Tubulin were used as loading controls.

(C) Bright field images of *Cited1*-overexpressing or control ESCs after treatment with DMSO or inhibitors for 3 days in CGR8 cells. LDN193189 (0.1 μM), Noggin (400 ng/mL) and K02288 (10 μM) are BMP inhibitors, and SB431542 (10 μM) is TGF-β inhibitor.

(D) qRT-PCR analyses of mRNA levels of trophoblast specific markers after transfection and treatment with inhibitors for 3 days in CGR8 cells. The average mRNA level in cells transfected with empty vector and treated with DMSO was set at 1.0. Data are shown as mean ± SD (n = 3). **p* < 0.05, ***p* < 0.01, ***p < 0.001.

**Figure S7. Related to Figure 6. Genomic DNA sequences before and after the gRNA-mediated cleavage and repair in *Bmpr2* loci.**

(A) Localization of exogenous Cited1 in E14T ESCs. E14T cells overexpressing *Cited1* were stained with anti-Cited1 antibody (red) and DAPI (blue).

(B) Genomic DNA sequences before and after the gRNA-mediated cleavage and repair in *Bmpr2* loci. Sanger sequencing data of a homogenous knockout line confirm the precise ligations of the double blunt ends for *Bmpr2*.

Table S1. Gene sets of TSC, TF, and *Oct4* KD

Table S2. DEGs of *Cited1* OE, *Cdx2* OE and *Gata3* OE

Table S3. Primers and gRNA or shRNA targeting sequences
