# Supplementary figures and images for: Transcription coactivator Cited1 acts as an inducer of trophoblast-like state from mouse embryonic stem cells through the activation of BMP signaling
Source: Cell Death Dis. 2018 Sep 11;9(9):924. doi: 10.1038/s41419-018-0991-1 (PMC6134011; doi:10.1038/s41419-018-0991-1)

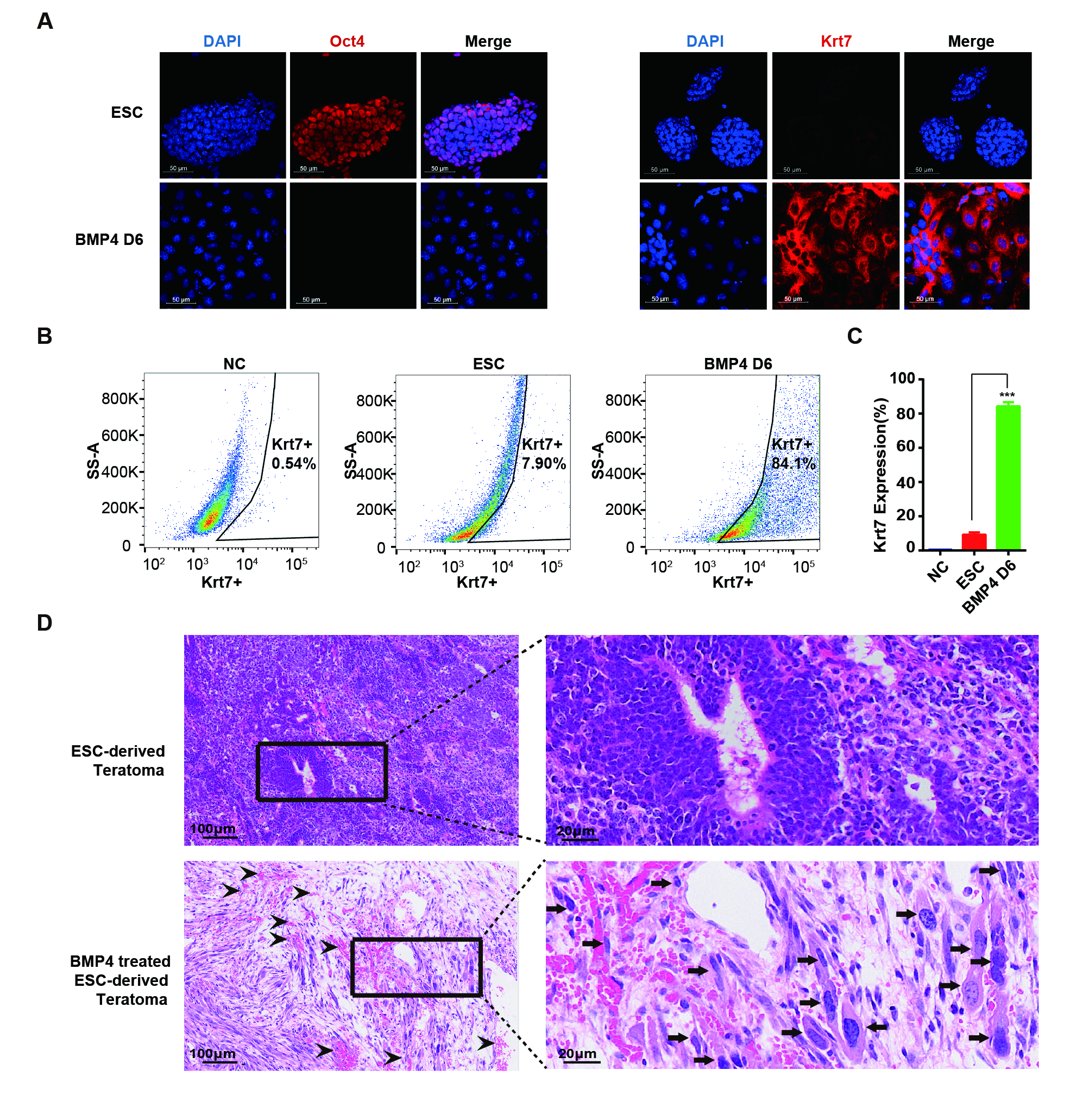

Supplement: Supplementary file 2 — Figure S1. Related to Figure 1. BMP4 induces mouse ESC differentiation with trophoblast genes up-regulation [file 41419_2018_991_MOESM2_ESM.tif]

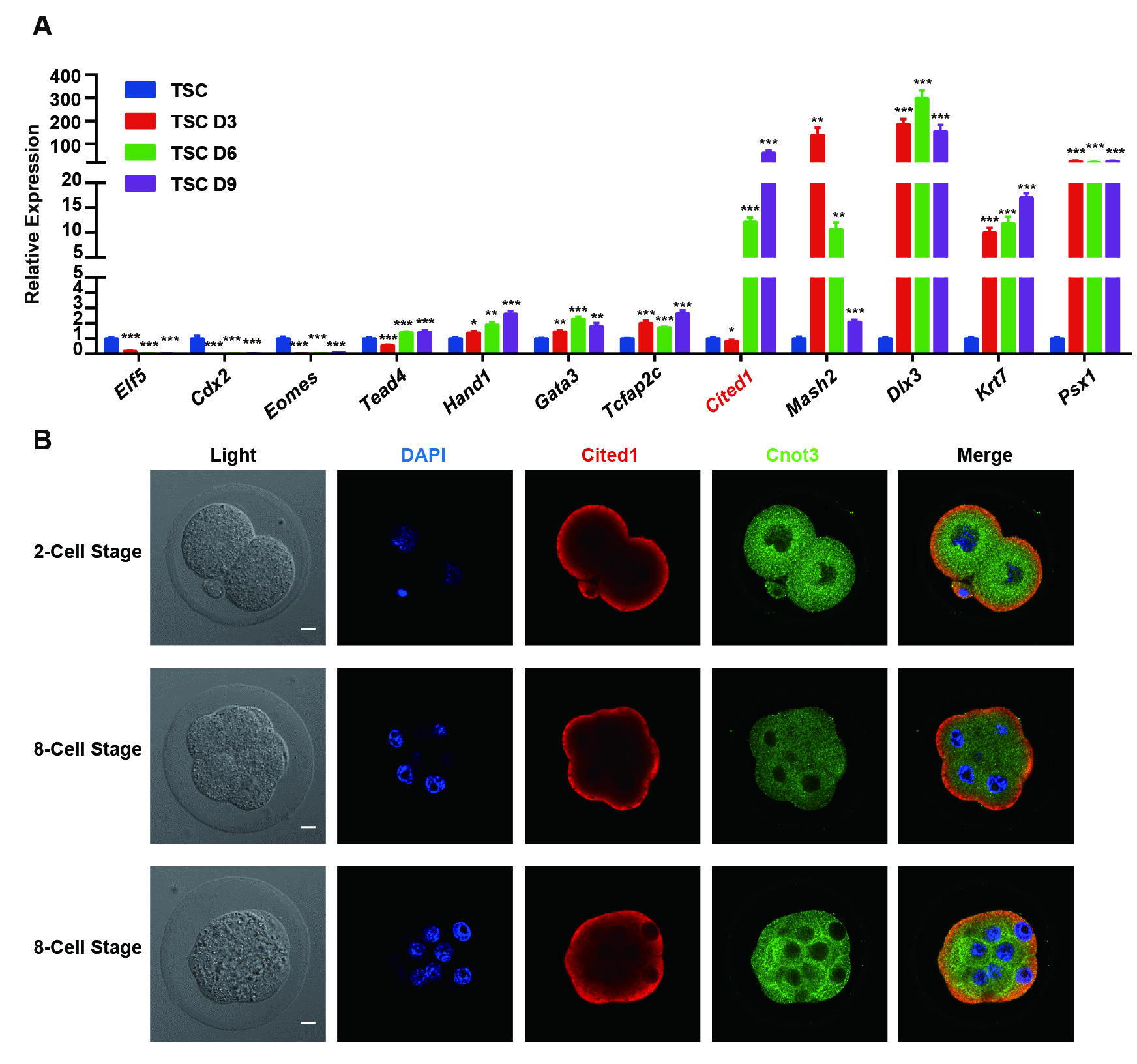

Supplement: Supplementary file 3 — Figure S2. Related to Figure 1. Cited1 is upregulated during TSC differentiation and Cited1 mainly expressed in the plasm of cells in early embryos [file 41419_2018_991_MOESM3_ESM.tif]

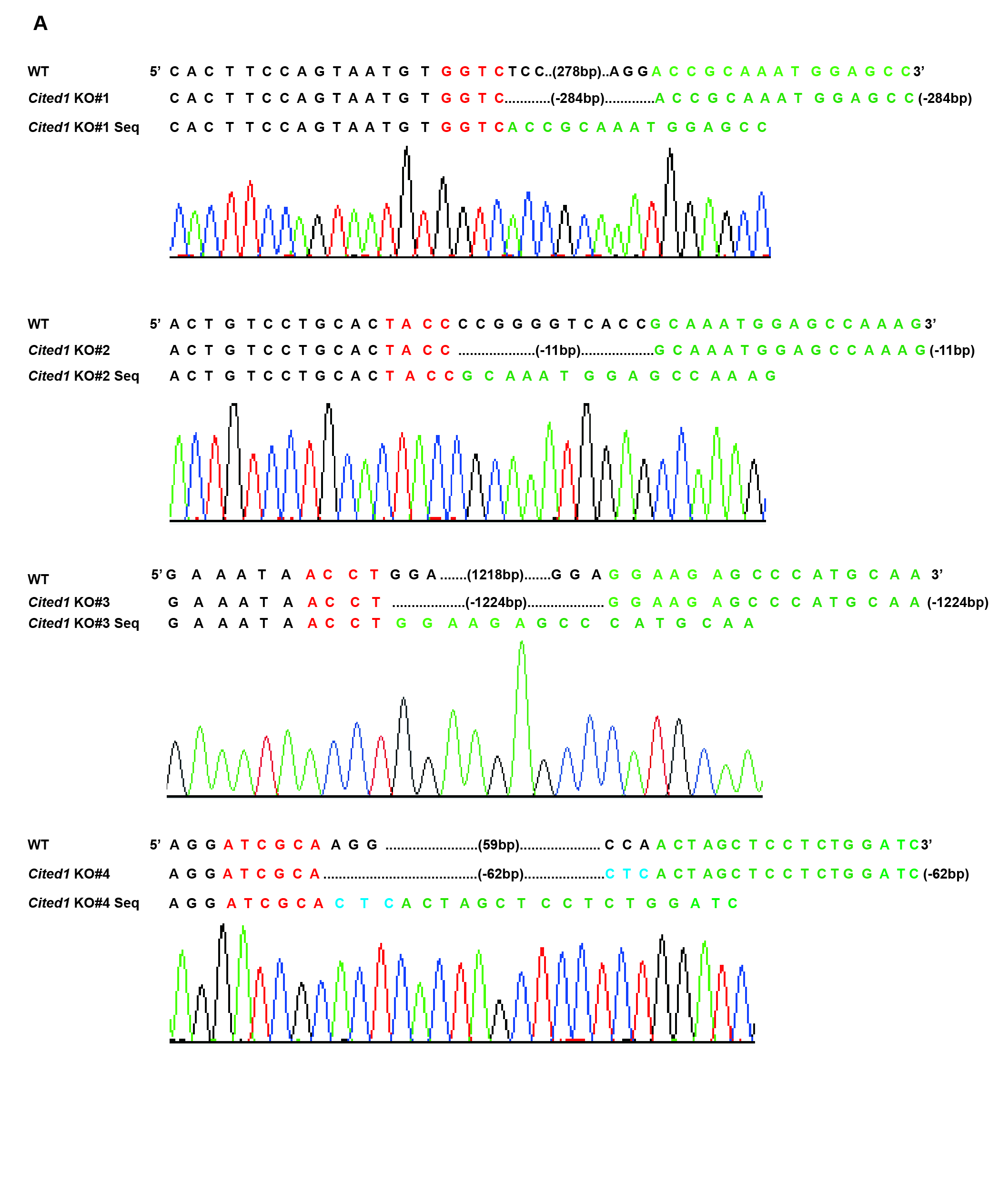

Supplement: Supplementary file 4 — Figure S3. Related to Figure 2. Genomic DNA sequences before and after the gRNA-mediated cleavage and repair in Cited1 loci [file 41419_2018_991_MOESM4_ESM.tif]

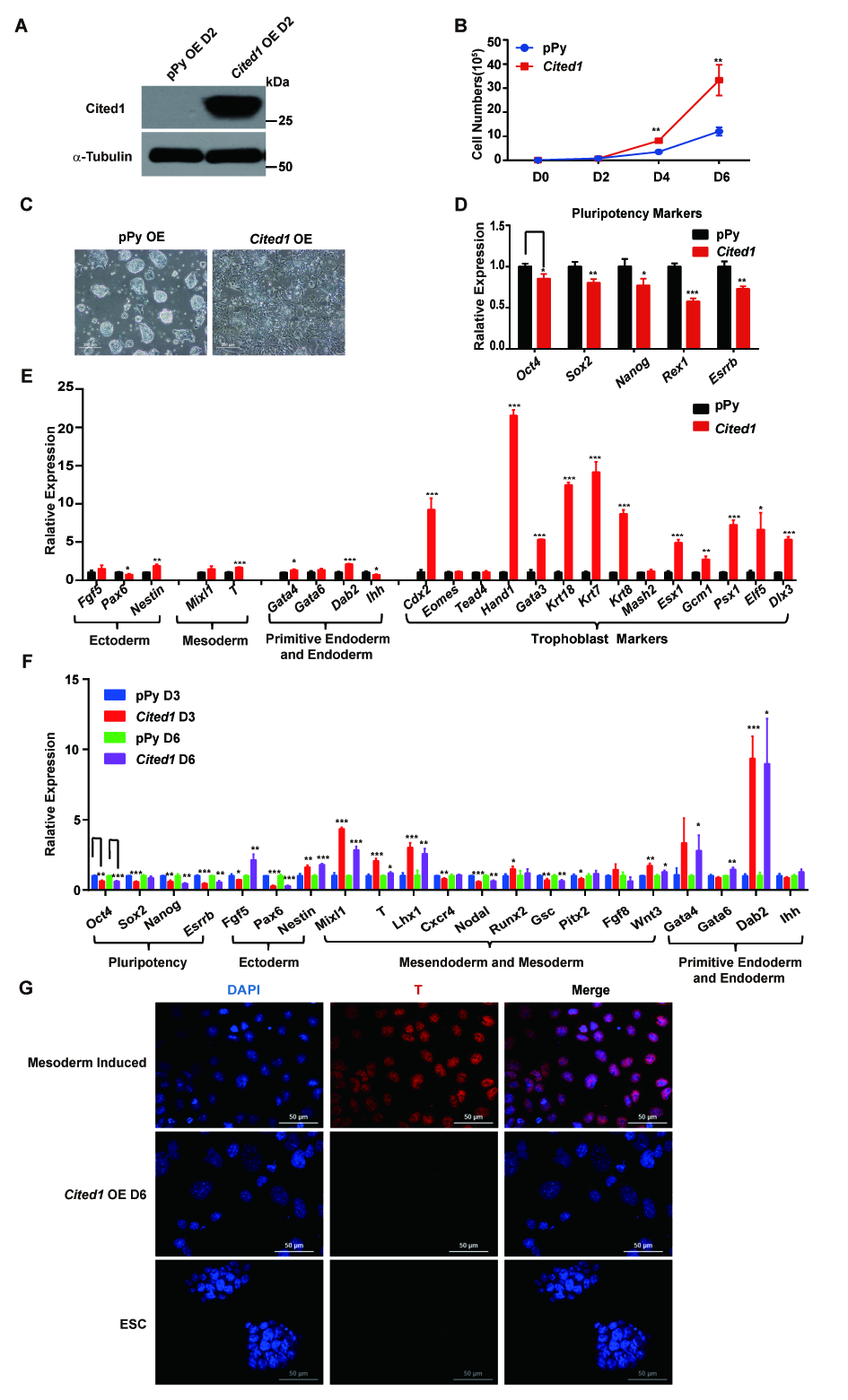

Supplement: Supplementary file 5 — Figure S4. Related to Figure 3. Forced expression of Cited1 promotes mouse ESC differentiation into trophoblast-like cells [file 41419_2018_991_MOESM5_ESM.tif]

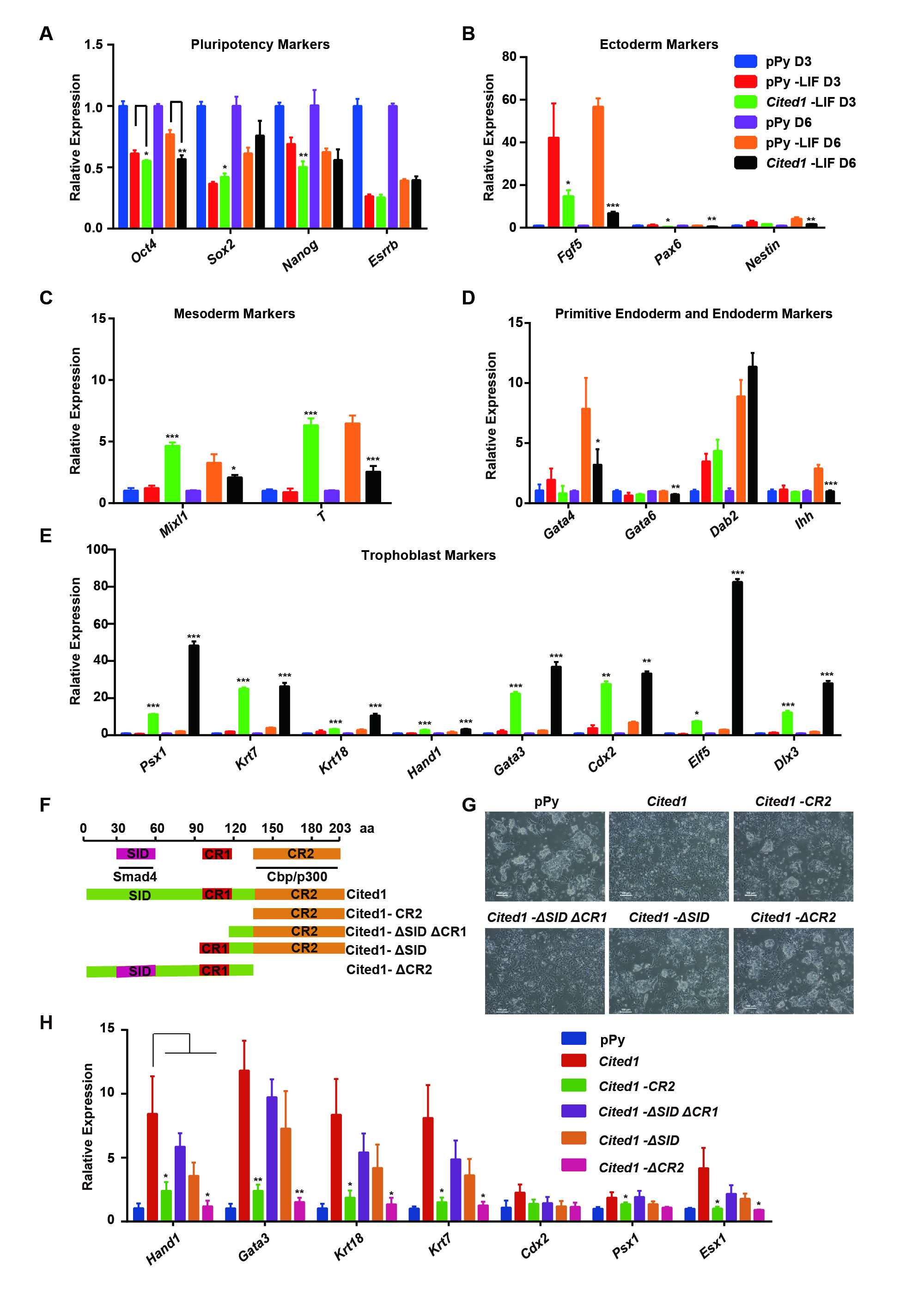

Supplement: Supplementary file 6 — Figure S5. Related to Figure 3. Ectopic Cited1 induces the expression of trophoblast markers under LIF withdrawal condition and the function of Cited1 depends on its full-length [file 41419_2018_991_MOESM6_ESM.tif]

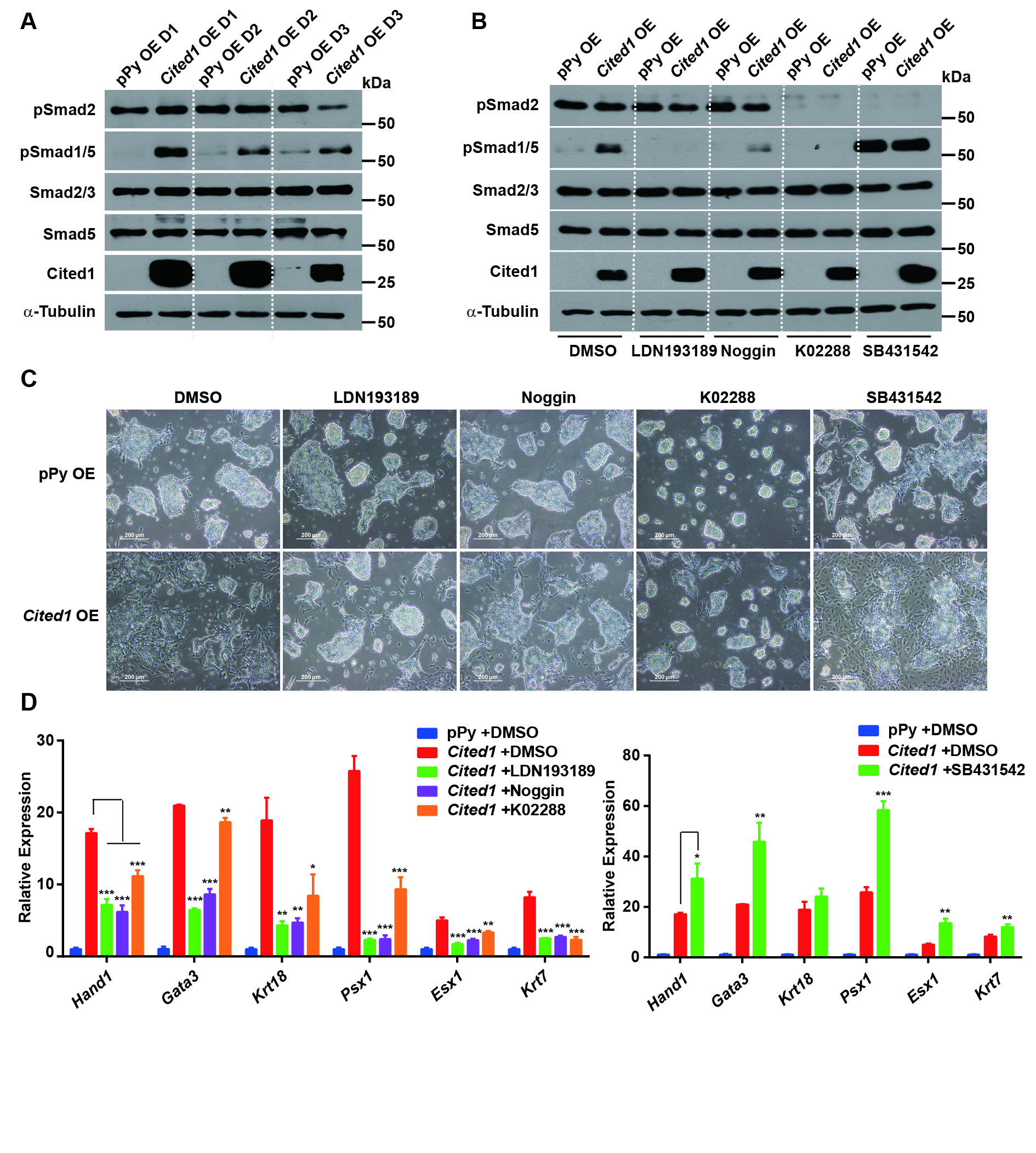

Supplement: Supplementary file 7 — Figure S6. Related to Figure 6. Inhibition of BMP signaling pathway partially rescues the differentiation phenotype caused by Cited1 overexpression in CGR8 cells [file 41419_2018_991_MOESM7_ESM.tif]

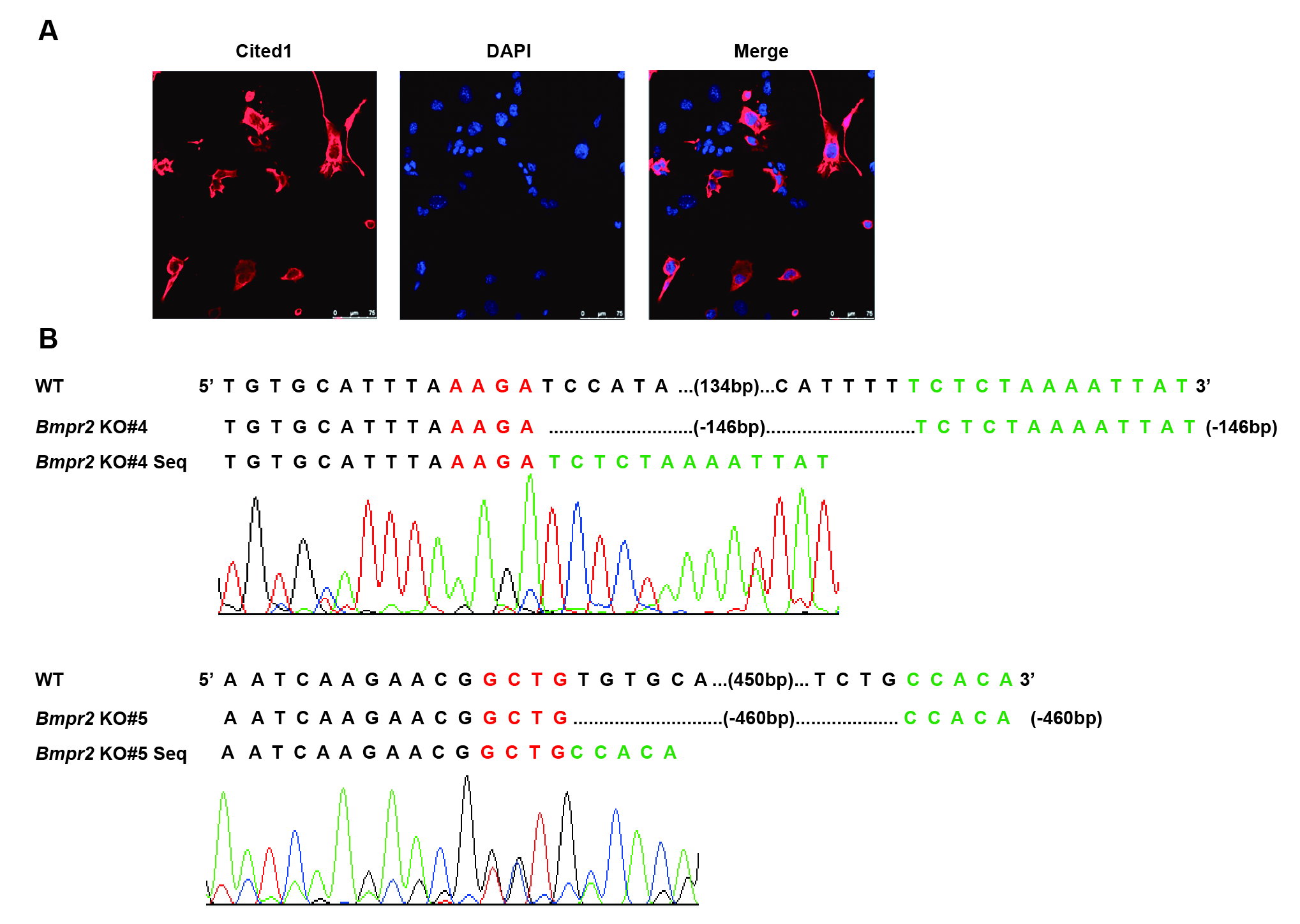

Supplement: Supplementary file 8 — Figure S7. Related to Figure 6. Genomic DNA sequences before and after the gRNA-mediated cleavage and repair in Bmpr2 loci [file 41419_2018_991_MOESM8_ESM.tif]
